# Developing and Applying RNA Empirical Models With Secondary Structure Insights for Orthoptera Phylogenetics

**DOI:** 10.1002/ece3.72068

**Published:** 2025-08-31

**Authors:** Huihui Chang, Yuan Huang, Lina Zhao, Qianqian Liu, Zhaohui Xie, Nian Liu

**Affiliations:** ^1^ College of Life Sciences and Engineering Henan University of Urban Construction Pingdingshan Henan China; ^2^ College of Life Sciences Shaanxi Normal University Xi'an Shaanxi China

**Keywords:** evolutionary model, orthoptera, phylogeny, RNA secondary structure

## Abstract

The evolutionary patterns exhibited by the ring and stem regions of the RNA secondary structure are distinct. Incorporating RNA secondary structure information into evolutionary models can improve the reliability of phylogenetic trees constructed using RNA sequences. However, empirically derived RNA evolutionary models remain scarce. In this work, we reconstructed conserved secondary structures (MPI > 60%, *z*‐score < 0) of 22 mitochondrial tRNAs and two rRNAs in Orthoptera, revealing stronger coevolution and selection signals in paired regions (*D* value < 0). By encoding sequences into 20‐character states and employing maximum likelihood estimation, we developed three substitution models: mtRNA16 (16‐state, pairing‐specific), mtRNA7 (7‐state, uniform mismatches), and mtRNA6 (6‐state, mismatch‐agnostic). All three models demonstrated robust performance, with the 16‐state model providing a better fit to the Orthoptera dataset than the other two models do. Phylogenetic analyses revealed that trees generated using these new models significantly outperformed those generated by a universal model, with the 16‐state model producing the most accurate phylogenetic relationships. The results underscore the advantages of incorporating RNA secondary structure information into evolutionary models. In particular, the 16‐state model offers enhanced precision in elucidating the phylogenetic relationships in Orthoptera, highlighting its potential for broader applications in RNA‐based phylogenetic studies.

## Graphical abstract

In the RNA secondary structure, paired regions experience stronger natural selection pressure than unpaired regions and are characterized by a higher density of coevolutionary sites. The empirical RNA evolutionary model that fully incorporates all base pairings can generate more reliable phylogenetic relationships. Empirical evolutionary models treating mismatched base pairs uniformly or completely disregarding mismatch information risk losing crucial phylogenetic signals.

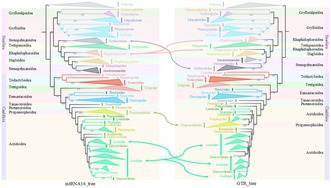

## 1 Introduction

An RNA secondary structure is a stem‐loop structure that is formed by the folding of an RNA single strand. This structure is composed of paired regions, known as stems (or helices), and unpaired regions, referred to as loops (Tahi et al. 2017). The stem region of RNA is primarily composed of Watson‐Crick (WC) base pairs (GC and AU), also referred to as standard base pairs. Although GU pairs are frequently observed in RNA structures and can form hydrogen bonds in RNA to create base pairs, they are less stable than GC and AU base pairs (Golden et al. 2020; Rousset et al. 1991). In addition, other pairs are relatively unstable and collectively called mismatches. The structural integrity of RNA has been highly conserved over long evolutionary periods, which is crucial for maintaining its functional capabilities (Savill et al. 2001). Secondary structure analysis of RNA sequences has been widely employed in taxonomic identification (Tang et al. 2017; Wang et al. 2015) and for inferring phylogenetic relationships among species (Akiyama and Sato 2023; Gesell and Schuster 2014; Golden et al. 2020; Meyer et al. 2019; Verma et al. 2020).

Natural selection tends to preserve the stability of secondary structures (Cheng et al. 2012; Muhire et al. 2014). When a mutation occurs in one of the base pairs in the stem region, disrupting the stability of the important functional structure, a compensatory mutation (compensatory base change, CBC) may occur in the complementary base. Such compensatory mutations may facilitate the reestablishment of structural stability and the restoration of pairing ability (Rousset et al. 1991). Phylogenetic studies on the basis of DNA sequences typically assume that sites evolve independently. However, the mechanism of compensatory base mutation in the RNA secondary structure differs from the conventional theory of DNA mutation on the basis of independent sites. The stem region, which is characterized by base pairing, and the loop region, which is characterized by base unpairing, may be subject to different evolutionary constraints (Nasrallah et al. 2011). WC complementarity between nucleotides is a fundamental aspect of the secondary structure of RNA. This complementarity serves as the foundation for ab initio secondary structure prediction methods derived from sequence alignments (Akiyama and Sato 2023). The robust coevolutionary pattern of WC pairs yields predictive signals, which emerge from compensatory mutations that restore fitness following a mutation at the interacting site on the opposing strand (Dutheil et al. 2010). This coevolution arises from an evolutionary constraint that limits substitution processes involving two or more positions in a molecule (Dutheil 2012; Pollock et al. 1999). From a phylogenetic standpoint, these sites undergo simultaneous substitutions or cosubstitutions. When phylogenies are constructed using the helical regions of RNA molecules, it is highly important to gain an understanding of how these parts of the sequences evolve to obtain reliable distance estimates.

Current RNA evolutionary models can be divided into two categories: theoretical and empirical. Similar to the DNA evolution model, theoretical models of RNA substitution also employ Markov processes to represent sequence changes. However, they necessitate modeling nucleotide pairing within stem regions as a distinct type of Markov chain. The number of states in RNA substitution models can vary, typically 6, 7, or 16, whereas the DNA evolution model is constrained to just 4 states. Notably, the DNA evolution model is applicable for describing the loop regions of RNA sequences since nucleotides in these areas are not restricted by pairing structures and are thus considered to evolve independently (Savill et al. 2001). The RNA 16‐state theoretical model employs a Markov process defined by a 16 × 16 rate matrix, encompassing all possible base pair combinations formed by the four nucleotides and specifying the relative transition rates between these states (Muse 1995; Schöniger and Von Haeseler 1994). In contrast, the 6‐state theoretical model focuses exclusively on the six canonical Watson‐Crick and wobble pairs (AU, GU, GC, UA, UG, CG, disregarding mispaired bases entirely Renée and Tillier 1994). The 7‐state model integrates ten mismatched base pairs into the MM to represent (G. Paul Higgs 2001; Tillier and Collins 1998). Subsequent parametrizations have produced families of nested models, including 7 A–7 F, 6 A–6 E, and 16 A–16 K (G. Paul Higgs 2001; P. G. Higgs 1998; Savill et al. 2001). Despite their theoretical sophistication, widespread adoption remains limited, hindered by over‐parameterization, weak identifiability, and the computational burden of high‐dimensional rate matrices.

Empirical RNA evolutionary models rely on substitution matrices obtained by comparing a substantial quantity of RNA sequence data (Smith et al. 2004; Subbotin et al. 2007). Smith et al. (2004) used secondary structure data from ribosomal RNA in bacterial and eukaryotic organisms. They transformed each sequence within the alignments into a sequence represented by a 20‐symbol code. By identifying ranges of sequence divergence, they derived substitution frequency matrices for the coded sequences. They also employed a technique initially devised for modeling amino acid substitutions to determine the actual evolutionary distance for each window, thus deriving a universal rate matrix (Smith et al. 2004). Empirical models offer several advantages, including ease of implementation; the code comprises only 20 symbols, facilitating incorporation into existing software for protein sequence analysis. Moreover, these models are useful for simulating the evolution of RNA sequences and structures simultaneously. Subbotin et al. (2007) applied comparable methods to plant‐parasitic nematode LSU genes, reducing erroneous phylogenetic support (Subbotin et al. 2007). Despite the aforementioned practical advantages of these empirical RNA evolutionary models, their substitution patterns may fail to accurately characterize mitochondrial RNA (mtRNA) evolution (Allen and Whelan 2014; Kosakovsky Pond et al. 2007), as they are predominantly derived from domain‐specific datasets with limited sampling.

Orthoptera encompasses over 25,700 extant species and represents one of the most diverse lineages within Polyneoptera insects (Cigliano et al. 2025; Grimaldi and Engel 2005). Convergent evidence from fossils, morphology, and molecular data strongly supports the monophyly of Orthoptera and its two suborders (Ensifera and Caelifera), yet internal relationships within these suborders remain incompletely resolved (Chang, Qiu, et al. 2020; Cigliano et al. 2025; Song et al. 2015, 2020; Zhou et al. 2017). Mitochondrial genomes (mitogenomes) have proven instrumental in elucidating Orthoptera phylogeny, resolving longstanding controversies in traditional morphological classification (Cameron 2014; Song et al. 2015). However, current research on the role of Orthoptera mitogenomes in phylogenetic analysis also has some limitations. Firstly, studies predominantly employ either single protein‐coding/rRNA genes or partial mitogenome concatenations for tree inference and population analyses (Cameron 2014; Chang et al. 2024; Chang, Qiu, et al. 2020; Zhang et al. 2023; Zhongying et al. 2020). Such fragmented datasets capture only subsets of biological signals, frequently generating conflicting topologies across Orthoptera lineages that impede phylogenetic resolution (Song 2018; Song et al. 2015). Furthermore, mitogenomic evolution involves nucleotide substitutions, indels, and structural rearrangements—processes constrained by functional demands of encoded products (proteins/RNAs) and exhibiting taxon‐specific evolutionary dynamics (Chang, Qiu, et al. 2020). However, the majority of contemporary mitogenome evolutionary analyses employ generic DNA or protein evolutionary models, with only a limited number of tailored evolutionary models for different taxonomic groups or molecules being applied to orthopteran phylogenetic analyses (Chang, Nie, et al. 2020). As the amount of data continues to increase, it is important to consider incorporating more diverse types of data into phylogenetic analysis, such as RNA secondary structure information. Furthermore, it is imperative to develop evolutionary models that are more adaptable to different data types to maximize information extraction from mitogenomic architecture to advance phylogenetic resolution.

Many phylogenetic analyses based on RNA fail to adequately consider the importance of RNA secondary structure information. Instead, they tend to rely on DNA evolutionary models for examining RNA sequence evolution (Chang et al. 2021, 2024; Liu et al. 2018; Ren et al. 2020; Shah et al. 2022; Song et al. 2015; Yue et al. 2023; Zhou et al. 2017). However, RNA models are more effective than DNA models in describing the sequence evolution of pairing regions (Allen and Whelan 2014; Kosakovsky Pond et al. 2007; Telford et al. 2005). It can therefore be concluded that partitioning sequences on the basis of secondary structure information and using RNA evolutionary models for the analysis of pairing region sequences may be a more suitable approach for the statistical analysis of mutation regularity in RNA sequences. Furthermore, the application of RNA evolutionary models may facilitate a more accurate description of the evolutionary relationships between species (Gesell and Schuster 2014; Hudelot et al. 2003; Telford et al. 2005). To date, empirical RNA evolutionary models remain scarce, and virtually all existing phylogenetic studies of Orthoptera that utilize mtRNA sequences have relied exclusively on DNA evolutionary models (Chang, Qiu, et al. 2020; Song et al. 2015; Zhou et al. 2017). Investigations incorporating RNA secondary structure information into dedicated empirical models are even rarer. Here, we characterize the secondary structures and selective regimes of orthopteran mtRNAs, construct a taxon‐specific empirical evolutionary model for these molecules, and evaluate its performance in reconstructing orthopteran phylogeny at the RNA level, thereby demonstrating the added value of such models for inferring evolutionary relationships. In this study, based on investigations of secondary structures, natural selection pressures, and coevolution of Orthoptera mtRNAs, we constructed a specific empirical evolutionary model and analyzed its application for reconstructing Orthoptera phylogenies at the RNA evolutionary level to demonstrate the value of such empirical models in inferring phylogenetic relationships.

## 2 Materials and Methods

### 2.1 DNA Extraction, Sequencing, and Mitogenome Assembly

The samples of *Eucriotettix amplifemurus* (Orthoptera: Tetrigoidea: Tetrigidae), *Paragavialidium curvispinum* (Orthoptera: Tetrigoidea: Tetrigidae), and *Tapiena bivittata* (Orthoptera: Tettigonioidea: Tettigoniidae) were collected from Jiangshan, Zhejiang, China (N28°44′42.14″, E118°37′2.51″) on August 4, 2018, with the voucher ID 18Z14; Xishuangbanna, Yunnan, China (N21°59′14.32″, E100°55′2.24″) on July 25, 2018, with the voucher ID 18Z15; and Xing'an, Guangxi, China (N25°37′19.37″, E110°25′32.11″) on July 8, 2009, with the voucher ID HY09A5. All the samples were preserved in 100% ethanol and stored in a −20°C freezer at the Institute of Zoology of Shaanxi Normal University. Genomic DNA was extracted from the muscle tissue of each sample using a DNeasy Blood and Tissue Kit (50‐QIAGEN 69504) and then stored at −20°C.

DNA from the three orthopteran species was sequenced using an Illumina HiSeq 2500 system, with a read length of 150 base pairs. The construction and sequencing of the DNA libraries were conducted by the Biomarker Company. NOVOPlasty v4.3.4 software (Dierckxsens et al. 2017) was used with the default parameters to facilitate mitogenome assembly. Transfer RNA sequences were identified using MITOS 2 (http://mitos.bioinf.uni‐leipzig.de/index.py) (Bernt et al. 2013), whereas the remaining genes were identified via Geneious Prime v2025.0.2 (Kearse et al. 2012) through a process of comparison with other related and reference mitogenomes, followed by manual verification. The mitogenome's structure was mapped using Proksee v2.0 (https://proksee.ca/) (Grant et al. 2023).

### 2.2 Construction of RNA Secondary Structure

A total of 292 orthopteran mitogenomes were utilized to construct the datasets, which included three newly determined and 289 previously published sequences from the National Center for Biotechnology Information (NCBI) database (Table S1). The sequences of 22 tRNA genes and 2 rRNA genes were extracted from the mitogenomes using PhyloSuite v1.2 (Zhang et al. 2020). Sequence alignments were performed using MAFFT v7.313 with the default settings, and the alignments of individual genes were concatenated using the PhyloSuite v1.2 program (Zhang et al. 2020).

A consensus sequence was obtained on the basis of the alignment results of each tRNA dataset. The general secondary structure mapping of tRNA was derived from the predicted results obtained through MITOS v2 (Bernt et al. 2013). In determining the general secondary structure of Orthoptera rRNA, we first referenced previously published Orthoptera rRNA secondary structures, including but not limited to *Acrida cinerea* (Liu and Huang 2010), *Gomphocerus sibiricus* (Zhang, Zhao, et al. 2013), *Sinopodisma pieli* (Zhongying et al. 2020), and *Longzhouacris mirabilis* (Zhang et al. 2023), etc., which served as the foundational framework for the universal RNA secondary structure of this order. Notably, most currently published RNA general structures within Orthoptera have been primarily constructed based on those of *Drosophila*, and these structures have gained widespread acceptance in the field (Cannone et al. 2002; Zhang, Zhao, et al. 2013; Zhongying et al. 2020). Furthermore, specialized comparative studies in the literature between Orthoptera and *Drosophila* RNA secondary structures provide a valuable reference (Liu and Huang 2010), facilitating our assessment of the accuracy of the Orthoptera general RNA secondary structure. Accordingly, we further validated the Orthoptera general RNA secondary structure using the rRNA secondary structures retrieved from public databases, the Comparative RNA Web (CRW) database (https://crw2‐comparative‐rna‐web.org/) (Cannone et al. 2002), specifically the small subunit ribosomal RNA (X54011) of *Drosophila teissieri* and the large subunit ribosomal RNA (X03240) of *Drosophila yakuba*. Finally, secondary structure predictions generated by MITOS v2 (Bernt et al. 2013) provided supplementary guidance (Table S2). Following a comprehensive analysis of the characteristics of orthopteran RNA sequences and the alignment results, the universal secondary structure of orthopteran RNA was ultimately determined. The dot‐bracket format was employed to calibrate the secondary structure of the consensus sequences. To ensure accurate correspondence between sequence sites and secondary structure positions, the comparison results of the dataset must be manually corrected. Ultimately, the general secondary structures of 22 transfer RNAs (tRNAs) and 2 ribosomal RNAs (rRNAs) were rendered using Forna (http://rna.tbi.univie.ac.at/forna/) (Kerpedjiev et al. 2015) and PseudoViewer v3 (Byun and Han 2009), respectively, on the basis of the consensus sequences and the secondary structures in dot‐bracket format. The composition and proportion of the different sites of each RNA sequence in 292 Orthopteran species were visualized using WebLogo v2.8.2 (http://weblogo.berkeley.edu/) (Crooks et al. 2004).

### 2.3 Secondary Structure Analysis

The minimum free energy (MFE) and structural conservation of each mtRNA were assessed by RNAz v2.1 (Gruber et al. 2011) (https://www.tbi.univie.ac.at/software/RNAz/). The tool provides several statistical metrics, including the structure conservation index (SCI), the *Z*‐score, which quantifies the MFE deviation from the mean of a set of randomized sequences with equivalent length and base composition, and the mean pairwise identity (MPI). A greater degree of conservatism in the secondary structure is associated with a higher SCI value. A negative *Z*‐score indicates a more stable secondary structure. If the MPI is less than 60%, simple sequence‐based methods are often unable to identify the optimal secondary structure. Additionally, RNAz was used to evaluate the probability estimate (*p* value) for the inclusion of thermodynamically stable and evolutionarily conserved RNA secondary structures. If the probability value (*p*) is greater than 0.5, the sequence is classified as functional RNA. A higher *p* value indicates increased confidence in this classification (Verma et al. 2020).

The 24 RNA datasets were classified into two categories, namely, paired and unpaired, on the basis of the overarching secondary structure of the RNA. Tajima's *D* test was conducted on each RNA gene's paired and unpaired region datasets using DnaSP v6 (Rozas et al. 2017) to assess the natural selection pressure across different datasets. A positive deviation from zero in the *D* value may indicate the influence of natural selection, with a greater deviation suggesting stronger selective pressure. Conversely, when *D* is equal to zero, it cannot be excluded that the neutral hypothesis is applicable (Muhire et al. 2014; Tajima 1989).

The coevolutionary sites in each RNA multiple sequence alignment dataset were analyzed using CoevRJ v1.2 (https://bitbucket.org/XavMeyer/coevrj/src/master/) (Meyer et al. 2019). The analysis of 22 tRNAs and 2 rRNAs was conducted over 1,000,000 to 2,000,000 generations, respectively, with the initial 25% of generations excluded as burn‐in. The remaining parameters were set to their default values.

### 2.4 Model Estimation

**FIGURE 1:**
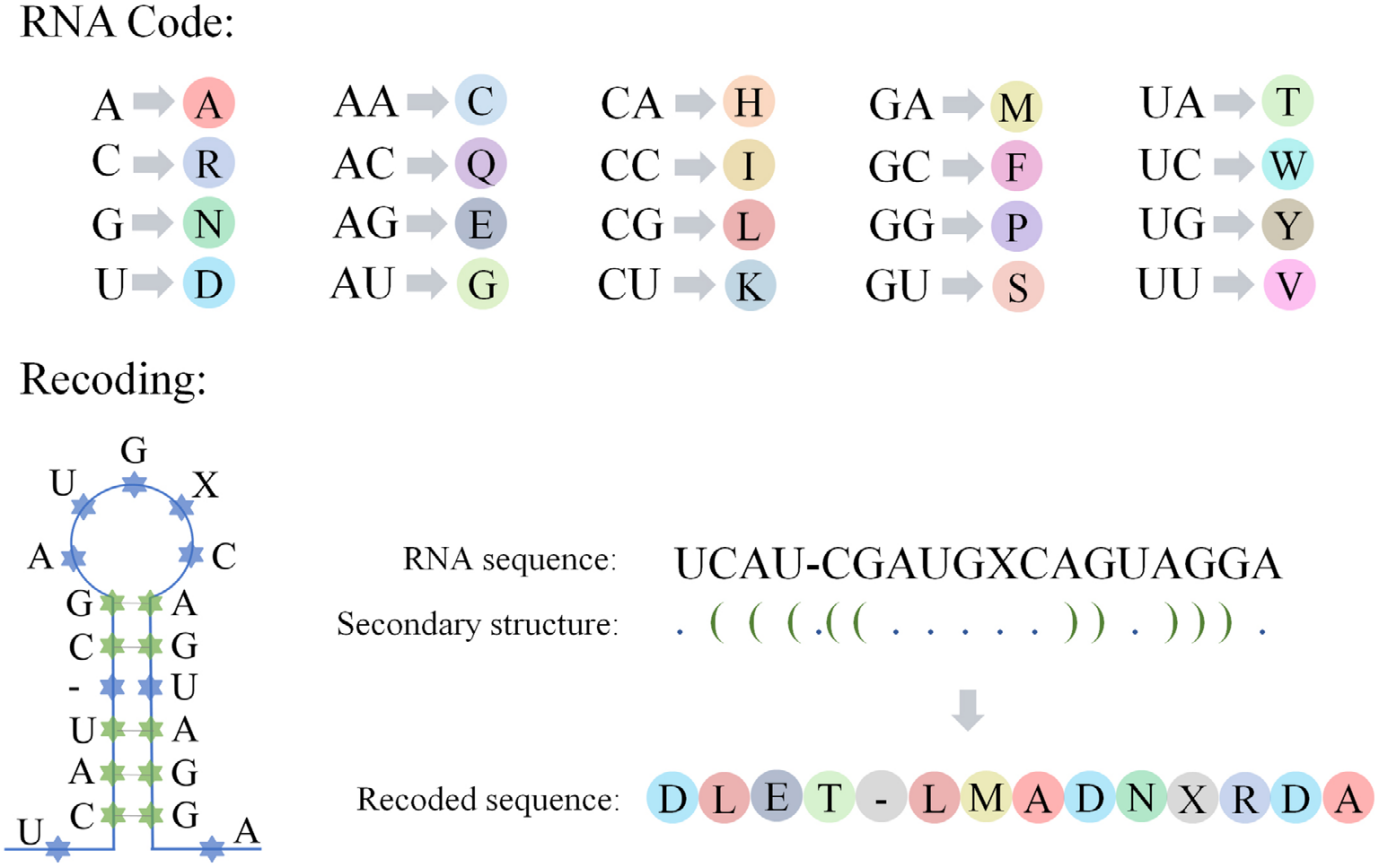
mtRNA sequence conversion. The RNA gene sequence is to be recoded in accordance with the encoding method that is displayed at the upper part of the figure. The newly encoded sequence contains base pairing information from the RNA secondary structure. The bottom of the figure presents a rudimentary illustration of the procedure for recording the RNA gene sequence, predicated on secondary structure information.

To incorporate information regarding the secondary structure of RNA into the new empirical evolution model, each sequence present in all the alignments was recorded by the coding pattern shown in Figure 1. This approach facilitated the conversion of coding symbols 4 to 20. First, the general secondary structure of each RNA was recorded in the form of a dot bracket on the next line of each sequence in the alignment, resulting in a new multisequence alignment file with three rows as a unit. The new multisequence alignment file was subsequently converted into a sequence file encoded by 20 symbols through the LongSeqDeal.java script (LongSeqDeal.java.txt). The resulting new coding file is in the conventional FASTA format, with the sequence name at the beginning of the sequence and the new sequence with 20 symbol codes.

The 24 encoded sequence files, comprising 292 Orthoptera species, were divided into two distinct files. One file included 263 species, whereas the other included 29 species, which were randomly selected. The former is used as the training dataset, and the latter is used as the test dataset. This allows for estimating the model replacement matrix from the larger training dataset to enhance the representativeness of the model parameters, and using the test dataset to evaluate the model's fit for data not included in the model construction. The alignment of each encoded sequence file was conducted using MAFFT v7.313 (Katoh et al. 2009). The concatenate sequence program in PhyloSuite v1.2 (Zhang et al. 2020) was employed to integrate the alignment training and test data into unified datasets, designated training and test datasets, respectively. The selection of the optimal model for the training dataset was conducted using ModelFinder v3.2 (Kalyaanamoorthy et al. 2017). The results indicated that Blosum62 was the most suitable model. Consequently, Blosum62 was selected as the initial model to estimate the empirical substitution matrix (the new model) using FastMG v1.0 (Chang, Nie, et al. 2020; Dang et al. 2014). The TreeBasedSplit was initially employed to partition the training dataset into subdatasets, with each subdataset containing data for up to 16 species (*k* = 16). The Estimate.pl program was subsequently employed to estimate the 20 × 20 empirical replacement matrix from the subset of the training dataset, resulting in a 16‐state empirical evolution model named mtRNA16. The creation of the 7‐state and 6‐state empirical evolution models necessitated the uniform processing of mismatches in the training dataset, with all mismatches being replaced by the letter “M” to indicate all mismatches in the new coding sequence. This process resulted in the creation of the 7‐state joint dataset. Conversely, substituting “M” in a 7‐state joint dataset with “‐” results in the creation of a 6‐state joint dataset. The same methodology was employed to obtain the subdatasets of the 7‐state and 6‐state training datasets. Given the distinctive attributes of the 7‐state and 6‐state datasets, the pertinent parameters of ConvertGrammar2Matrix.perl, as outlined in the FastMG v1.0 methodology (Dang et al. 2014), were appropriately calibrated to facilitate model parameter estimation. The empirical evolution models for the 7‐state and 6‐state models were estimated using the mtRNA16 model as the initial model. These models were designated mtRNA7 and mtRNA6, respectively.

### 2.5 Model Analysis

The RNA sequence files, comprising 292 Orthoptera, 93 Ensifera, and 199 Caelifera species, were reencoded, after which the encoded sequence files were aligned using MAFFT v7.313 (Katoh et al. 2009). The resulting datasets were then concatenated. The processing followed the principles of mismatched coding symbols, resulting in the generation of 16‐state, 7‐state, and 6‐state datasets comprising 292, 93, and 199 species, respectively. The 16‐state, 7‐state, and 6‐state empirical evolution models of the three datasets were subsequently estimated by FastMG v1.0 (Dang et al. 2014). The empirical replacement models constructed with the 16‐state, 7‐state, and 6‐state datasets containing 292, 93, and 199 species, respectively, were designated with the corresponding species numbers. For example, the three models constructed with the 292 species datasets were designated mtRNA16_292, mtRNA7_292, and mtRNA6_292. The robustness of the mtRNA16, mtRNA7, and mtRNA6 models was evaluated using model parameters constructed from different datasets. Pearson correlation analysis of parameters between different models was performed using IBM SPSS Statistics 20.

The test dataset, comprising 29 species, was randomly divided into three subdatasets using the RandomSplit.pl function in FastMG with *k* = 8, as recommended by Dang et al. (2014). The performance of three models, mtRNA16, Blosum62, and mtOrt (Chang, Nie, et al. 2020), was then evaluated based on the 16‐state test dataset using IQ‐TREE v2.2 (Nguyen et al. 2015). Initially, the site log‐likelihood and AIC values were calculated for each sub‐dataset using a variety of models. The suitability of the mtRNA16 model for the test dataset was evaluated on the basis of the mean site log‐likelihood and AIC values. Similarly, the mismatches in the test dataset were coded, resulting in the creation of two new test datasets, one with seven states and one with six states. The same method was then employed to obtain subdatasets of the 7‐state and 6‐state test datasets, which were subsequently evaluated to assess the performance of mtRNA7, mtRNA16, and mtOrt based on these datasets. Finally, the fitting of mtRNA7 and mtRNA6 to the test dataset was evaluated, and the discrepancies in the fitting of the test dataset for mtRNA16, mtRNA7, and mtRNA6 were compared.

### 2.6 Phylogenetic Analysis

The objective of this study was to explore the differences in the phylogenetic relationships of Orthoptera on the basis of different models and to evaluate the performance of various RNA empirical evolution models. Six species from other insect groups were selected as outgroups, and 292 Orthoptera species were chosen as intergroups (Table S1). New coding RNA datasets comprising 16‐state, 7‐state, and 6‐state sequences, as well as RNA gene sequence datasets and 13 protein sequence datasets derived from the mitogenomes of 298 species, were constructed. Phylogenetic trees were constructed with mtRNA16, mtRNA7, mtRNA6, and GTR models, based on the 16‐state, 7‐state, 6‐state, and RNA gene datasets, respectively. These trees were designated mtRNA16_tree, mtRNA7_tree, mtRNA6_tree, and GTR_tree accordingly. Additionally, an extra tree (mtOrt_tree) was constructed using the mtOrt model in combination with the 16‐state dataset. For all five models (mtRNA16, mtRNA7, mtRNA6, GTR, and mtOrt), among‐site rate heterogeneity parameters (proportion of invariant sites [+I] and gamma‐distributed rates [+G4]) were optimized (e.g., mtRNA16 + I + G4), whereas the remaining parameters were set to their default values. The 13 protein sequence datasets were aligned using MAFFT v7.313 (Katoh et al. 2009), and a joint dataset of protein sequences was subsequently constructed. The optimal model for this dataset was determined using ModelFinder with default parameters (based on BIC values) (Kalyaanamoorthy et al. 2017), which indicated that the mtOrt+R10 model provided the best fit. The phylogenetic tree of Orthoptera derived from mtOrt+R10 and the protein sequence dataset was proposed to represent an optimal tree (Best_tree) that closely approximates the true evolutionary relationships. An additional comparison was subsequently conducted between Best_tree and the other phylogenetic trees. All phylogenetic trees were constructed using IQ‐TREE v2.2 (Nguyen et al. 2015), which employs the maximum likelihood method with 1000 bootstraps and 1000 SH‐aLRT branch tests. The discrepancies in likelihood values and node support among phylogenetic trees generated by various models were evaluated to assess the impact of parameter discrepancies across different trees. To analyze the variations in topological structures, the matching split distance (MS) and Robinson–Foulds distance (RF) metrics were employed to measure the distance between phylogenies by TreeCmp v2.0 (Bogdanowicz et al. 2012). The MS distance is sensitive to the branch length, while the RF distance only considers the topological structure. All distances are based on the normalized average distance obtained from the Yule model (Bogdanowicz et al. 2012). This is because the Yule model can better simulate the actual process of biological evolution. The smaller the distance, the smaller the difference between the trees.

## 3 Results

### 3.1 Three New Mitogenomes

The mitogenomes of *E. amplifemurus*, *P. curvispinum*, and *T. bivittata* are 18,337 bp, 18,117 bp, and 15,086 bp in length, respectively (Figure S1). All three are characterized as closed circular molecules (GenBank accession numbers PQ524196, PQ524197, and PQ389431, respectively). The complete mitogenomes of *E. amplifemurus* and *P. curvispinum* contain a conserved set of 37 genes, including 13 protein‐coding genes (PCGs), large and small rRNAs (*rrnL* and *rrnS*), 22 transfer RNAs (tRNAs), and a large noncoding region known as the A+ T‐rich region or control region. The mitogenome of *T. bivittata* presents an identical number and arrangement of genes to those of the first two species, except for *trnY*. To verify the absence of *trnY* in the future, the HMMER 3.1b2 (Eddy 2011) was utilized to scan the entire genome of *T. bivittata* using the HMM profiles of *trnY* from all species. In the context of the default E‐value threshold (E < 10), it was observed that no significant matches were detected. In future studies, additional samples and data will be provided to validate the *trnY* data in the mitochondrial genome of *T. bivittata* . An arrangement order translocation of *trnK* and *trnD* (KD rearrangement) is found in *E. amplifemurus* and *P. curvispinum* but not in *T. bivittata* .

### 3.2 Secondary Structure of mtRNAs

**FIGURE 2:**
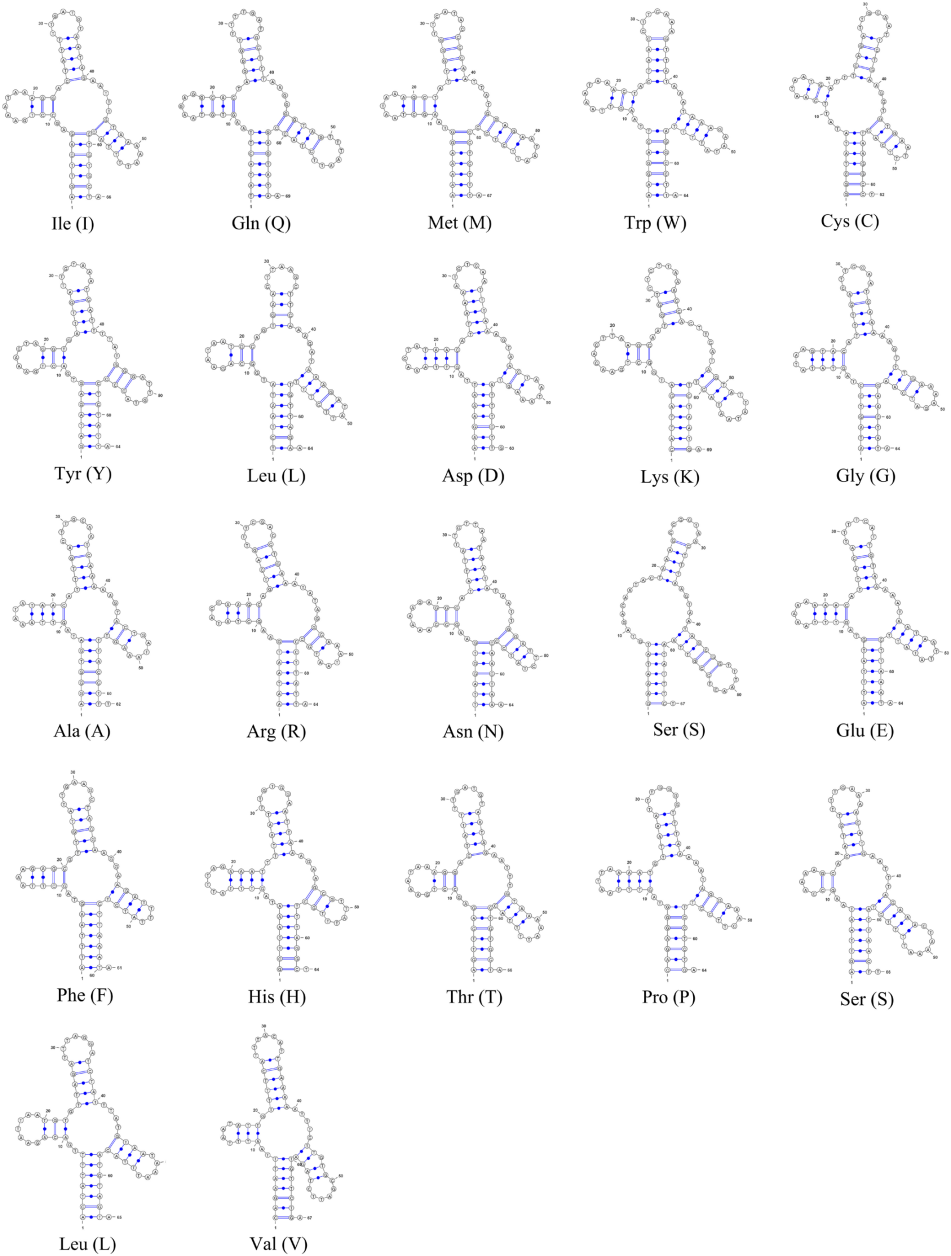
The general secondary structures of 22 tRNAs in mitochondrial genomes of Orthoptera insects represented by the sequences of Paragavialidium curvispinum. The number of horizontal lines in the pairing area indicates the number of hydrogen bonds between bases. All the tRNA genes presented a typical clover‐leaf secondary structure, except trnSAGN, which lacks the dihydrouridine (DHU) arm.

**FIGURE 3:**
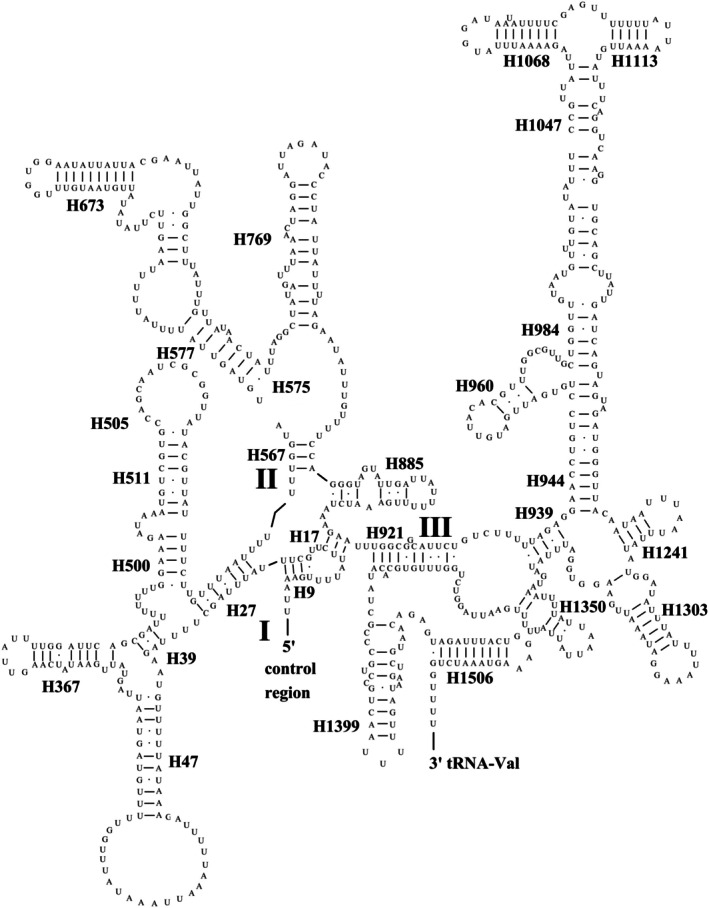
The general secondary structure of rrnS in mitochondrial genomes of Orthoptera insects represented by the sequence of Paragavialidium curvispinum. The horizontal lines in the pairing area represent typical Watson‐Crick base pairs (GC and AU), while the dots indicate base mismatches.

**FIGURE 4:**
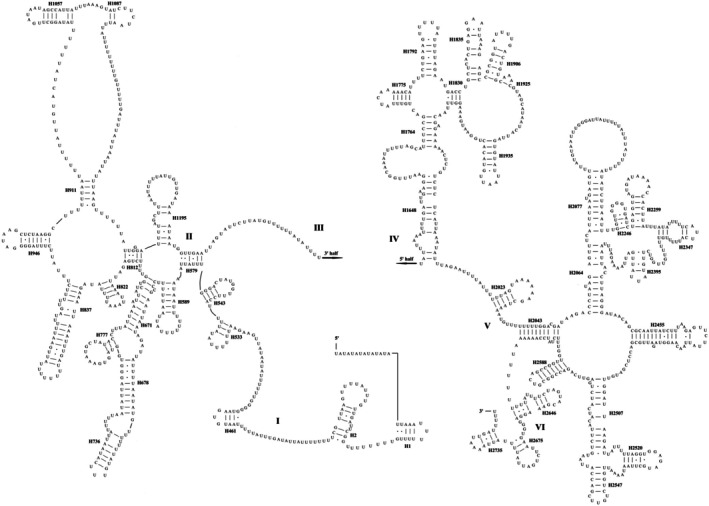
The general secondary structures of rrnL in mitochondrial genomes of Orthoptera insects represented by the sequence of Paragavialidium curvispinum. The horizontal lines in the pairing area represent typical Watson‐Crick base pairs (GC and AU), while the dots indicate base mismatches.

The universal secondary structure of RNA is based on the consistent sequence and the published secondary structure of mitochondrial RNA of Orthoptera species as the framework, and was established after verification with the published data in the Comparative RNA Web (CRW) database and the prediction results of MITOS v2 (Bernt et al. 2013) (Figures S2–S4 and Table S2). Given that the length of the consensus sequence derived from the multiple sequence alignment exceeds that of the actual RNA sequences, we constructed representative secondary structures for Orthoptera mtRNAs using the *P. curvispinum* sequence for more intuitive visualization (Figures 2, 3, 4). All the tRNA genes presented a typical clover–leaf secondary structure, except *trnS* ^*AGN*^, which lacks the dihydrouridine (DHU) arm. The secondary structure of *rrnS* is characterized by three major domains (I, II, and III), whereas that of *rrnL* is distinguished by six major domains (I, II, III, IV, V, and VI).

### 3.3 Conservation Analysis of Secondary Structure

**FIGURE 5:**
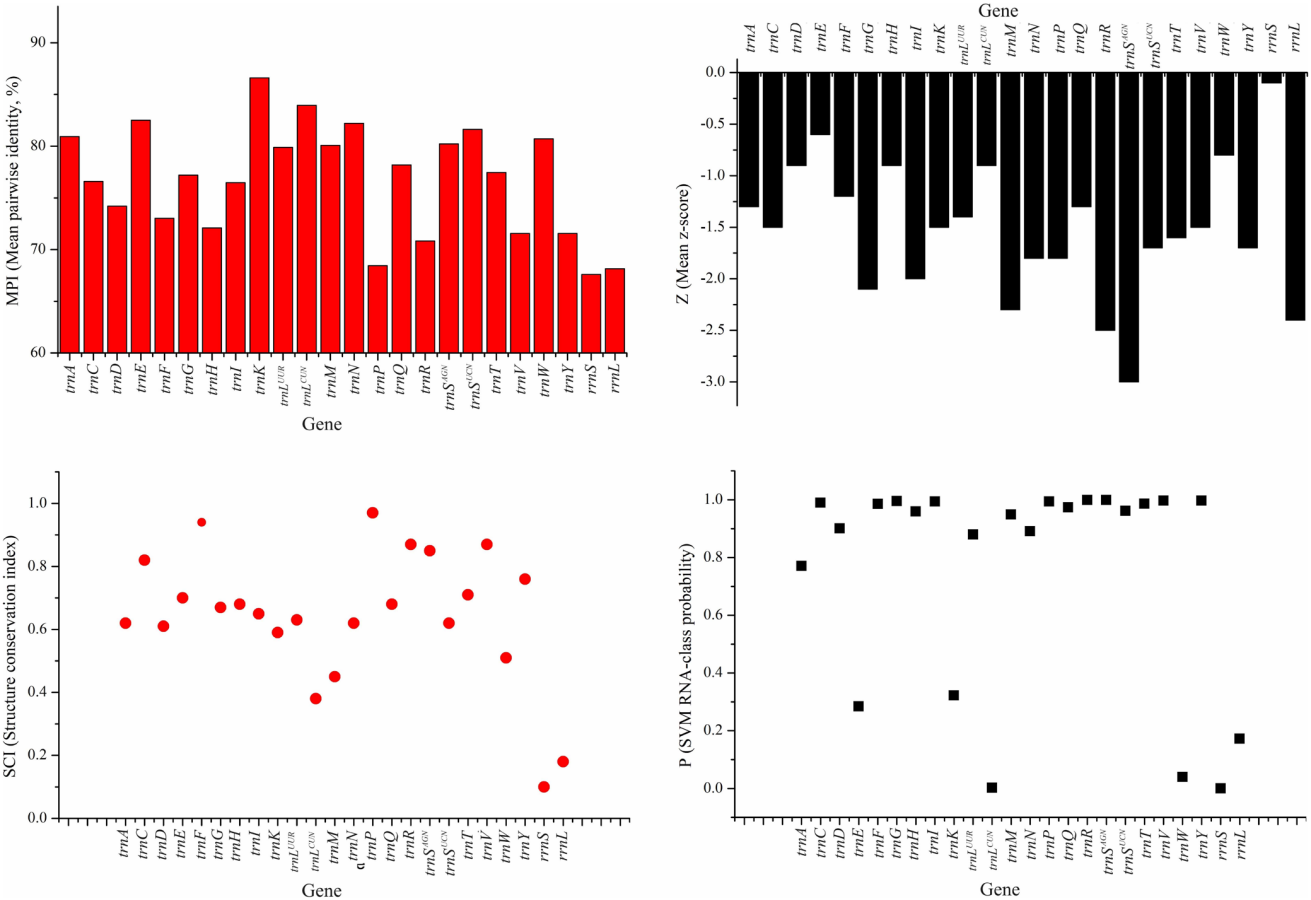
The conservation of mtRNAs secondary structure. The top left corner shows the mean pairwise identity (MPI), and if the MPI is less than 60%, simple sequence‐based methods are often unable to identify the optimal secondary structure. The bottom left corner shows the structure conservation index (SCI), and a greater degree of conservatism in the secondary structure is associated with a higher SCI value. The top right corner shows the *Z*‐score, which quantifies the MFE deviation from the mean of a set of randomized sequences with equivalent length and base composition; the larger the *Z*‐score, the greater the deviation. The bottom right corner shows the probability estimate (*p*‐value) for the inclusion of thermodynamically stable and evolutionarily conserved RNA secondary structures. If the probability value (*p*) is greater than 0.5, the sequence is classified as functional RNA. A higher *p* value indicates increased confidence in this classification.

The MPI values for all mtRNAs ranged from 67.6% (*rrnS*) to 86.6% (*trnK*), with all values exceeding 60%. This finding indicates that simple sequence‐based secondary structure prediction methods are capable of identifying the optimal general secondary structure. The z‐scores ranged from −3 (*trnS* ^*AGN*^) to −0.1 (*rrnS*), with all values falling below zero, indicating a relatively stable sequence structure. The SCI values range from 0.1 (*rrnS*) to 0.97 (*trnP*), indicating that the tRNA secondary structure is more conserved than that of rRNA. The *p* values varied between 0.0000 (*rrnS*) and 0.9992 (*trnS* ^*AGN*^). Except for two rRNAs, *trnE*, *trnK*, *trnL* ^*CUN*^, and *trnW*, the *p* values of the remaining tRNAs were all greater than 0.5, indicating that the secondary structures of the majority of RNAs are highly reliable (Figure 5).

### 3.4 Natural Selection Pressure and Coevolution Analysis

Tajima's *D* test was applied to 24 mtRNA datasets, revealing that the majority of both paired and unpaired regions presented *D* values less than 0 (Table 1). This finding suggests a pattern consistent with that of purifying selection, except for the paired region of *trnY*. In 14 out of the 24 datasets, the *D* value for paired regions exceeded that for unpaired regions. Four datasets (*trnE*, *trnK*, *trnN*, and *trnS* ^*UCN*^) exhibited significant (*p* < 0.05) deviations from zero in their paired regions, whereas only one dataset (*trnS* ^*UCN*^) demonstrated such a deviation in its unpaired region.

**TABLE 1:** Tajima's *D* statistics for paired and unpaired sites in mtRNAs secondary structure.

Gene	Paired		Unpaired	
	Tajima's *D*	*p*	Tajima's *D*	*p*
*trnA*	−1.29434	*p* > 0.10	−0.94447	*p* > 0.10
*trnC*	−1.33552	*p* > 0.10	−1.39649	*p* > 0.10
*trnD*	−1.35739	*p* > 0.10	−0.76015	*p* > 0.10
*trnE*	−2.04138	*p* < 0.05*	−1.34195	*p* > 0.10
*trnF*	−0.63138	*p* > 0.10	−0.523	*p* > 0.10
*trnG*	−1.35739	*p* > 0.10	−1.30912	*p* > 0.10
*trnH*	−0.69789	*p* > 0.10	−0.60008	*p* > 0.10
*trnI*	−1.68622	0.10 > *p* > 0.05	−1.22307	*p* > 0.10
*trnK*	−1.79255	*p* < 0.05*	−1.00557	*p* > 0.10
*trnL* ^*UUR*^	−1.00193	*p* > 0.10	−0.97018	*p* > 0.10
*trnL* ^*CUN*^	−1.54817	0.10 > *p* > 0.05	−1.16487	*p* > 0.10
*trnM*	−1.18498	*p* > 0.10	−1.50007	*p* > 0.10
*trnN*	−1.78129	*p* < 0.05*	−0.60337	*p* > 0.10
*trnP*	−0.11819	*p* > 0.10	−0.97307	*p* > 0.10
*trnQ*	−0.89305	*p* > 0.10	−0.86276	*p* > 0.10
*trnR*	−0.89127	*p* > 0.10	−1.74477	0.10 > *p* > 0.05
*trnS* ^*AGN*^	−1.3311	*p* > 0.10	−1.79022	*p* < 0.05*
*trnS* ^*UCN*^	−1.87474	*p* < 0.05*	−0.90696	*p* > 0.10
*trnT*	−1.15278	*p* > 0.10	−1.19369	*p* > 0.10
*trnV*	−1.20403	*p* > 0.10	−0.59128	*p* > 0.10
*trnW*	−0.95405	*p* > 0.10	−1.52352	*p* > 0.10
*trnY*	0.06667	*p* > 0.10	−1.09502	*p* > 0.10
*rrnS*	−0.97726	*p* > 0.10	−1.29817	*p* > 0.10
*rrnL*	−0.87068	*p* > 0.10	−1.46201	*p* > 0.10

* A Tajima's D value that significantly deviates from zero (*p* < 0.05) suggests that the null hypothesis of neutral evolution can be rejected.

All the mtRNA datasets revealed a diverse set of coevolutionary sites, the majority of which are located within the paired regions of the secondary structure (Table 2, Figures S2–S4). In the 22 tRNA datasets, all coevolutionary sites in *trnN*, *trnQ*, and *trnS* ^*UCN*^ were found to be located within the paired region (100%). In contrast, the lowest coevolution site in other tRNAs that appeared in the paired region was found in *trnS* ^*AGN*^ (71.9%). The percentage of coevolutionary sites relative to the total length of the multisequence alignment across the tRNA datasets ranged from 16.9% (*trnW*) to 50.7% (*trnF*). The proportion of coevolutionary sites within the paired region in the two rRNA datasets was lower than that observed for tRNAs, ranging from 64.6% (*rrnS*) to 56.8% (*rrnL*), respectively. In addition, their proportions relative to the total alignment lengths were 20.0% (*rrnS*) and 20.2% (*rrnL*), respectively (Table 2).

**TABLE 2:** Coevolution site in mtRNAs sequences.

Gene	Number of coevolution sites	Number of coevolution site in paired region	Proportion of coevolution sites in paired regions	Total proportion of coevolution sites
*trnA*	20	19	0.950	0.270
*trnC*	23	22	0.957	0.299
*trnD*	24	23	0.958	0.312
*trnE*	24	21	0.875	0.308
*trnF*	37	33	0.892	0.507
*trnG*	28	27	0.964	0.354
*trnH*	27	25	0.926	0.351
*trnI*	41	35	0.854	0.488
*trnK*	25	18	0.720	0.329
*trnL* ^*UUR*^	29	24	0.828	0.387
*trnL* ^*CUN*^	26	23	0.885	0.351
*trnM*	24	23	0.958	0.300
*trnN*	14	14	1.000	0.175
*trnP*	38	31	0.816	0.447
*trnQ*	26	26	1.000	0.371
*trnR*	32	30	0.938	0.386
*trnS* ^*AGN*^	32	23	0.719	0.457
*trnS* ^*UCN*^	28	28	1.000	0.364
*trnT*	24	21	0.875	0.304
*trnV*	22	20	0.909	0.289
*trnW*	14	12	0.857	0.169
*trnY*	20	18	0.900	0.244
*rrnS*	175	113	0.646	0.200
*rrnL*	303	172	0.568	0.202

### 3.5 Datasets and New Models

The newly encoded 16‐state, 7‐state, and 6‐state datasets, generated via multiple sequence alignment, have lengths of 4458 bp, 4895 bp, and 2054 bp, respectively. To composition, the three newly encoded datasets exhibit analogous patterns. The nucleotide compositions of the four bases in the unpaired region are similar across the three datasets (16‐state/7‐state/6‐state): A (0.12/0.12/0.21) > U (0.10/0.10/0.19) > G (0.03/0.03/0.05) > C (0.02/0.02/0.03). Among the six typical WC base pairs (GC, CG, AU, UA, GU and UG), the proportions are as follows: (0.09/0.09/0.16) > UA (0.08/0.08/0.15) > GC (0.05/0.05/0.09) > CG (0.03/0.03/0.05) > UG (0.02/0.02/0.03) > GU (0.01/0.01/0.03). In the 16‐state dataset, UU has the highest proportion of inconsistencies (0.20), followed by AA (0.16), whereas other inconsistencies have smaller proportions ranging from 0.001 to 0.006. The ratio of MM in the 7‐state dataset combining all the mismatches is 0.46.

**FIGURE 6:**
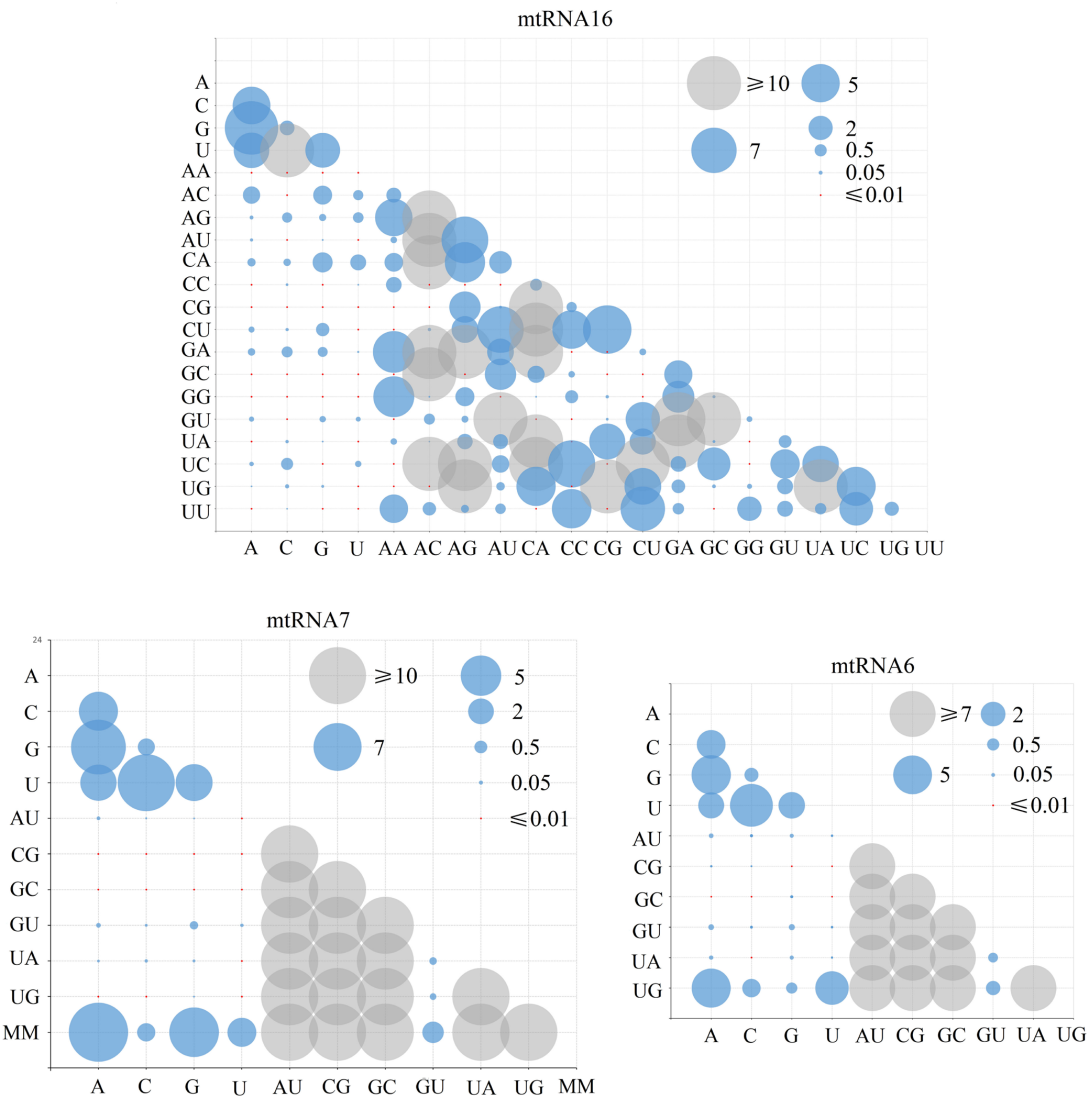
Exchangeability rates of three mtRNA empirical substitution models. The size of the bubbles (circles) in the figure represents the magnitude of the replacement rate. The blue bubbles represent the original data, the red bubbles represent the replacement rate less than 0.01, and the gray bubbles represent the replacement rate greater than 10 or 7.

The alternative rate matrices of the three empirical evolution models, namely, mtRNA16, mtRNA7, and mtRNA6, were estimated according to the training dataset. These matrices were configured as follows: 4 × 4 + 16 × 16 (20 × 20), 4 × 4 + 7 × 7 (11 × 11), and 4 × 4 + 6 × 6 (10 × 10), respectively (Figure 6). The 4 × 4 matrix represents the substitution rates between single‐strand bases in unpaired regions, whereas the remaining matrices reflect the substitution rates between paired bases in different states. In the unpaired region of the three models (mtRNA16, mtRNA7, and mtRNA6) (4 × 4), the highest substitution rate among the four bases was observed between the UC pairs, followed by the GA pairs, whereas the GC pairs presented the lowest rate. Among the six typical WC base pairs (6 × 6), the highest exchange rate was observed between AU–GU in all three models, followed by UG–CG. However, the minimum exchange rates varied between these models: GC–CG for mtRNA16, UG–GU for mtRNA7, and UA–GU for mtRNA6. This pattern was also observed within mismatched pairs in the mtRNA16 model, where UC–AC had a notably elevated replacement rate, and multiple mismatched base pair replacements were recorded as zero for combinations such as CC–AG and UC–GG (Figure 6 and Table S3).

### 3.6 Robustness of the New Models and Their Fit to the Test Dataset

The 16‐state substitution matrix (mtRNA16, mtRNA16_292, mtRNA7_93, and mtRNA16_199), which was constructed from different datasets, demonstrated a high correlation between the frequency vector and the substitution rate, with highly significant correlations (correlation coefficient close to 1) (Table S4). Furthermore, no significant differences (*p* > 0.05) were observed between the model parameters, indicating that the mtRNA16 model parameters are robust. Highly significant correlations were also found for the frequency vectors and most of the substitution rates of the different 7‐state models (mtRNA7, mtRNA7_292, mtRNA7_93, and mtRNA7_199); however, such correlations were not observed for the substitution rates of mtRNA7_93 and mtRNA7_292 (correlation coefficients different from 1), suggesting substantial differences between these two matrices (Table S4). Significant differences between the parameters of different 7‐state matrices were only observed with the mtRNA7_93 and mtRNA7_199 models (*p* < 0.05), whereas no significant differences were detected with the other models (*p* > 0.05). Significant correlations were identified between the mtRNA7 model and the other three replacement matrices (*p* < 0.01). While the correlation coefficient was not as high as that observed for mtRNA16, it nevertheless indicates that the mtRNA7 model demonstrates a certain degree of robustness. For all of the 6‐state replacement matrices (mtRNA6, mtRNA6_292, mtRNA6_93, and mtRNA6_199), highly significant correlations are found in terms of frequency vectors (*p* < 0.01); however, in terms of replacement rates, highly significant correlations (*p* < 0.01) were only found using the mtRNA6, mtRNA6_292, and mtRNA6_199 models, whereas no significant correlation was observed between mtRNA6_93 and the other matrices (*p* > 0.05) (Table S4). No significant differences were observed between the model parameters (*p* > 0.05), suggesting that the mtRNA6_93 parameters are significantly different from those of the other models, whereas mtRNA6 shows some robustness but is not universally applicable to all datasets.

**FIGURE 7:**
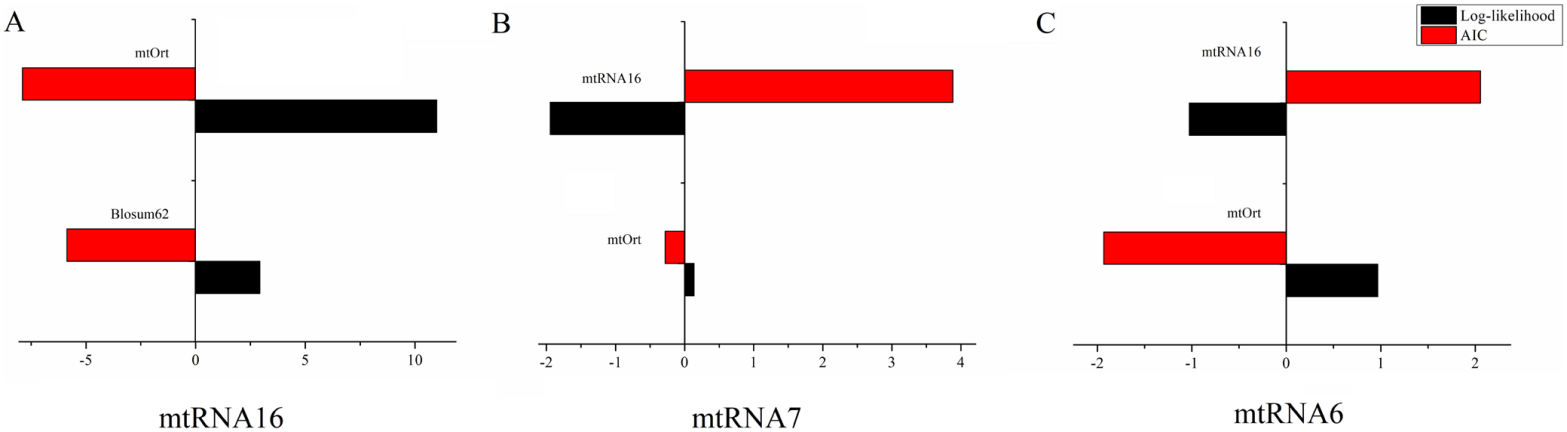
The mean difference of log‐likelihood and AIC scores of per site between different models on testing data sets. The negative value of the AIC difference between the models indicates that the model on the *x*‐axis is superior to the one on the *y*‐axis. The positive value of the log‐likelihood difference indicates that the model on the *x*‐axis is superior to the one on the *y*‐axis. (A) The mean differences between the mtRNA16 model and the mtOrt and Blosum62 models. (B) The mean differences between the mtRNA7 model and the mtOrt and mtRNA16 models. (C) The mean differences between the mtRNA6 model and the mtOrt and mtRNA16 models.

For the test data set composed of data that were not involved in the modeling process, both mtRNA7 and mtRNA6 achieved superior average site log‐likelihood and Akaike information criterion (AIC) values in comparison to those of the mtOrt model. Furthermore, mtRNA16 exhibited superior performance in comparison with that of both mtRNA7 and mtRNA6 based on their respective 7‐state and 6‐state datasets (Figure 7).

### 3.7 Phylogenetic Analysis of Orthoptera

The analysis results of the site log‐likelihood values indicate that among the six trees (mtRNA16_tree, mtRNA7_tree, mtRNA6_tree, GTR_tree, mtOrt_tree, and Best_tree), there were six pairs of trees (mtRNA6_tree‐mtRNA7_tree, mtOrt_tree‐mtRNA6_tree, mtOrt_tree‐mtRNA16_tree, mtRNA6_tree‐Best_tree, mtRNA16_tree‐Best_tree, and mtOrt_tree‐Best_tree) where no significant differences in site log‐likelihood values were found (*p* > 0.05). In contrast, all other pairwise comparisons exhibited significant differences (*p* < 0.05). The absolute values of the correlation coefficients between site log‐likelihoods were generally low, ranging from 0.004 to 0.470. Notable exceptions were the high correlations observed between mtRNA16_tree and mtGTR_tree (0.960, *p* < 0.01) and between mtOrt_tree and mtGTR_tree (0.960, *p* > 0.05) (Table S5). All six trees showed significantly different bootstrap values (*p* < 0.05), which were positively correlated with extremely high significance (0.928–0.994, *p* < 0.01). Regarding branch lengths, only the comparisons between mtOrt_tree and mtGTR_tree and between mtRNA16_tree and Best_tree revealed no significant differences (*p* > 0.05), while all other comparisons were significantly different (*p* < 0.05). Although some trees exhibited significant correlations in branch lengths (*p* < 0.05), the absolute values of these correlation coefficients remained low (0.002–0.284) (Table S5).

Topological distance analysis revealed that, under the MS distance metric, the largest topological distance was between Best_tree and mtRNA7_tree (0.322), and the smallest was between mtRNA16_TREE and mtGTR_TREE (0.151). When compared to the optimal tree, the smallest topological distance was between mtRNA16_tree and Best_tree (0.181). Under the RF distance metric, the largest topological distance was between Best_tree and mtRNA6_tree (0.361), and the smallest was between mtRNA16_TREE and mtGTR_TREE (0.193). Again, when compared to the optimal tree, the smallest topological distance was between mtRNA16_tree and Best_tree (0.263) (Table S5).

**FIGURE 8:**
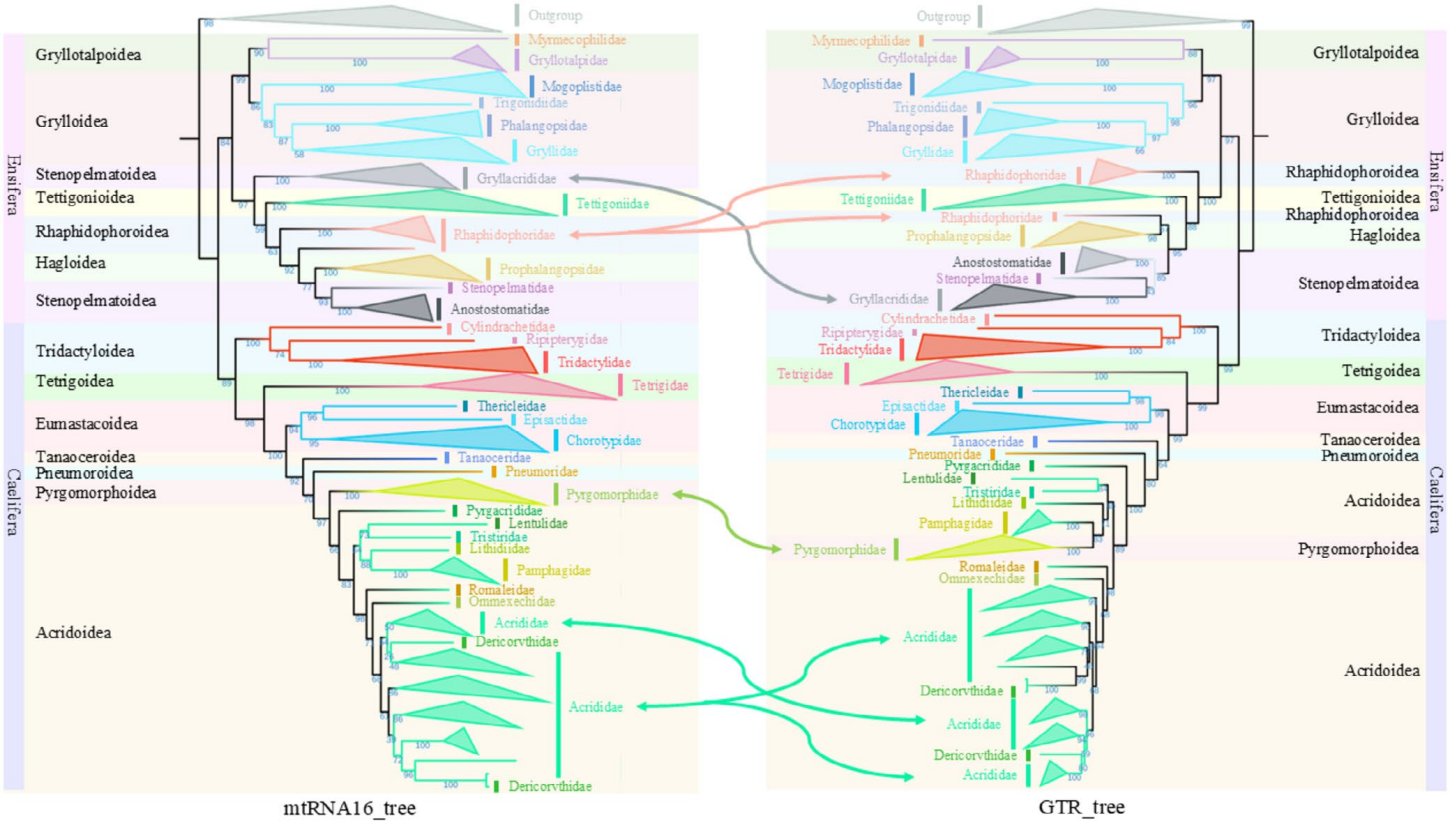
The phylogenetic relationships among the higher taxa of Orthoptera in mtRNA16_tree and GTR_tree. The leftmost/rightmost branches of the two trees, the background color, and the color of the branches represent the superfamilies. Different families are distinguished by different colors. The arrows between the two trees indicate the branches where the two trees diverge, and the arrow colors represent different families.

From a tree topology perspective, at the suborder level, only the mtRNA16_tree, mtRNA6_tree, GTR_tree, and Best_tree can divide Orthoptera into two sister groups, Ensifera and Caelifera (Figure 8, Figures S5–S8). In contrast, mtRNA7_tree and mtOrt_tree grouped the Grylloidea and Gryllotalpoidea clades of Ensifera to Caelifera (Figures S7 and S8). In four of the six trees analyzed, except mtRNA7_tree and mtRNA6_tree, the Ensifera species were grouped into two clades, Grylloidea and Gryllotalpoidea (Grylloid), with these two superfamilies forming sister groups. Two families of Gryllotalpoidea, Myrmecophilidae, and Gryllotalpidae, are sister groups to each other in five trees (except mtRNA7_tree). The superfamily Grylloidea appeared as four families, the relationships among which in mtRNA16_tree, Best_tree, and GTR_tree are as follows: (Mogoplistidae + (Trigonidiidae + (Phalangopsidae + Gryllidae))). However, in both mtRNA7_tree and mtRNA6_tree, these four families did not cluster into a single clade. Although the four families were grouped in mtOrt_tree, Mogoplistidae and Trigonidiidae formed sister groups.

Another clade of Ensifera (nongrylloid) comprises four superfamilies whose relationships in mtRNA16_tree are as follows: ((((Stenopelmatoidea + Hagloidea) + Rhaphidophoroidea) + Tettigonioidea) + Stenopelmatoidea). The Rhaphidophoroidea species did not cluster. Conversely, Best_tree displayed these relationships as ((((Stenopelmatoidea + Hagloidea) + Tettigonioidea) + Rhaphidophoroidea) + Stenopelmatoidea), with all Rhaphidophoroidea species grouped on a single branch. mtRNA6_tree grouped *Troglophilus neglectus* from Rhaphidophoroidea with species from Hagloidea, whereas other members of Rhaphidophoroidea formed sister groups to Tettigonioidea. In GTR_tree, the relationships between the four superfamilies were represented as follows: ((((Stenopelmatoidea + (Hagloidea + Rhaphidophoroidea)) + Tettigonioidea) + Rhaphidophoroidea)). Importantly, the familial relationships within these superfamilies mirrored those observed at the superfamily level; however, there were variations between different phylogenetic trees.

In total, seven families were represented within Caelifera. In mtRNA6_tree, Tetrigoidea was positioned at the base of Caelifera. In the other five trees, Tridactyloidea occupied the base of Caelifera, whereas Tetrigoidea formed a sister group with a clade comprising five superfamilies, excluding Tridactyloidea. The relationships between the three families included in Tridactyloidea were as follows in mtRNA16_tree, Best_tree, and GTR_tree: (Cylindrachetidae + (Tridactylidae + Ripipterygidae)); however, in mtRNA6_tree, mtRNA7_tree, and mtOrt_tree, the relationships were as follows: (Tridactylidae + (Cylindrachetidae + Ripipterygidae)). Pyrgomorphoidea was grouped with Pamphagidae in mtRNA7_tree and GTR_tree, but in the other trees, Pyrgomorphoidea and Acridoidea were sister groups to each other and were the last to diverge within Caelifera. In mtRNA16_tree, Best_tree, and GTR_tree, Pneumoroidea was closely related to Pyrgomorphoidea, whereas Eumastacoidea was distantly related to Pyrgomorphoidea. However, a close relationship between Tanaoceroidea and Pyrgomorphoidea was found in mtOrt_tree. Conversely, in mtRNA6_tree and mtRNA7_tree, the three families within Eumastacoidea did not cluster into a single clade, and the relationships among Eumastacoidea, Pneumoroidea, and Tanaoceroidea became obscured. Within Acridoidea, the relationships between families varied considerably between trees, yet two species of Dericorythidae were consistently identified within Acrididae across all the tree analyses. In the other five trees, Tridactyloidea occupied the base of Caelifera, whereas Tetrigoidea formed a sister group with a clade comprising five superfamilies, excluding Tridactyloidea. The relationships between the three families included in Tridactyloidea were as follows in mtRNA16_tree, Best_tree, and GTR_tree: (Cylindrachetidae + (Tridactylidae + Ripipterygidae)); however, in mtRNA6_tree, mtRNA7_tree and mtOrt_tree, the relationships were as follows: (Tridactylidae + (Cylindrachetidae + Ripipterygidae)). Pyrgomorphoidea was grouped with Pamphagidae in mtRNA7_tree and GTR_tree, but in other trees, Pyrgomorphoidea and Acridoidea were sister groups to each other and were the last to diverge within Caelifera. In mtRNA16_tree, Best_tree, and GTR_tree, Pneumoroidea was closely related to Pyrgomorphoidea, whereas Eumastacoidea was distantly related to Pyrgomorphoidea. However, a close relationship between Tanaoceroidea and Pyrgomorphoidea was found in mtOrt_tree. In mtRNA6_tree and mtRNA7_tree, the three families within Eumastacoidea did not cluster into one clade, and the relationships among Eumastacoidea, Pneumoroidea, and Tanaoceroidea were unclear. In Acridoidea, relationships between families varied considerably between trees, but two species of Dericorythidae appeared within Acrididae in all tree analyses.

## 4 Discussion

### 4.1 The Universal Secondary Structure Analysis of mtRNAs

During the process of constructing the universal secondary structures of RNAs, we found that the variations in tRNA sequence length are attributable primarily to the TΨC arm/ring and the DHU loop (Figure 2 and Figure S2). The deletion of the DHU arm in *trnS* ^*AGN*^ is considered a distinctive feature of the mitogenomes of metazoans and is also prevalent in Orthoptera (Liu and Huang 2010; Wolstenholme 1992). Secondary structural abnormalities are rarely observed in other tRNAs (Zhongying et al. 2020).

The universal secondary structures of the mitochondrial *rrnS* and *rrnL* genes in Orthoptera are consistent with the models of other insects presented on the CRW website and those of other insects that have been published (Cannone et al. 2002; Do et al. 2006; Lan et al. 2023; Lian et al. 2022; Liu and Huang 2010) (Figures 3, 4, Figures S3 and S4). For example, compared with those of *Arma custos* (Insecta: Hymenoptera), *Arma custos* (Insecta: Heteroptera), *D. teissieri*, and *D. yakuba* (Insecta: Diptera), the secondary structures of *rrnS* and *rrnL* in Orthoptera species add only a few pairs in part of the helix region without altering the overall number or configuration of helices (Cannone et al. 2002; Gillespie et al. 2006; Hickson et al. 1996; Lian et al. 2022). The secondary structure of *rrnS* is notably conserved, with the three major domains present in species such as *E. coli* and *Drosophila* (Clary and Wolstenholme 1987). However, in insects, the secondary structure of *rrnS* is simplified (Cannone et al. 2002; Gillespie et al. 2006; Lan et al. 2023; Lian et al. 2022; Liu and Huang 2010). For example, the secondary structure of *rrnS* in Orthoptera is considerably simplified in domains I and II in comparison to that observed in *E. coli* (Figure 3 and Figure S3) (Hickson et al. 1996). Domain III of *rrnS* is relatively less simple and more conserved in insects (Carapelli et al. 2004; Liu and Huang 2010). In insects, the secondary structure of mitochondrial *rrnL* is much simpler than that of *E. coli* , with the most notable difference being the absence of domain III (Cannone et al. 2002; Gillespie et al. 2006). This simplification is also observed in Orthoptera insects (Figure 4 and Figure S4) (Lan et al. 2023; Liu and Huang 2010). Furthermore, considerable variations in the domain near the 5′ end have been observed (Gillespie et al. 2006; Lian et al. 2022; Misof and Fleck 2003), with a particularly pronounced simplification observed in domain I. In Orthoptera, domain I is similar to that of *Drosophila* but retains only a few helical structures when contrasted with that of *E. coli* (Liu and Huang 2010).

Owing to the significant variation in the primary sequence of domain II, various models have been proposed, each making distinct assumptions about this region. However, the secondary structure of this domain in insects is notably similar (Cannone et al. 2002; Gillespie et al. 2006; Lan et al. 2023; Lian et al. 2022). Domains IV and V, near the 3′ end, show relatively high degrees of conservation and evolutionary conservation and have been used for phylogenetic analysis in previous studies (Liu and Huang 2010; Misof and Fleck 2003). Domain VI is also significantly simplified in insects and is described differently in *rrnL* secondary structure models of different insect taxa (Misof and Fleck 2003), with only three helices conserved in Orthoptera (Figure 4 and Figure S4).

To further ensure accuracy, we constructed universal Orthoptera RNA secondary structures using published rRNA secondary structures from Orthoptera (*Oedaleus decorus decorus*, OP627272) (Lan et al. 2023), Drosophila (the mall subunit ribosomal RNA (X54011) of *D. teissieri* and the large subunit ribosomal RNA (X03240) of *D. yakuba*) (Caetano‐Anolles 2002) and Heteroptera (*Arma custos*, NC_051562) (Lian et al. 2022) as frameworks, respectively, and conducted comparative analyses using ViennaRNA v2.0 (Hofacker 2003). Results showed that the two rRNA universal structures derived from Orthoptera frameworks were identical to our universal structures (with a secondary structure distance of zero), validating the accuracy of the current universal Orthoptera RNA secondary structures.

### 4.2 Evaluation of the Universal Secondary Structure of mtRNAs

The secondary structures of all 24 mtRNAs presented common features (MPI > 60%, *Z* < 0), indicating a relatively stable and conserved structure, with high confidence levels (most mtRNAs had *p* values greater than 0.5) (Figure 5). In comparison, rRNAs demonstrate a lower degree of conservation relative to that of tRNAs (the SCI values of 22 tRNAs were all greater than 0.2, and the SCI values of 2 rRNAs were less than 0.2). This difference is attributable to the comparatively uncomplicated nature of the secondary structure of tRNAs, which predominantly adopt a cloverleaf structure. Consequently, variations in the secondary structure among different species are largely confined to differences in base composition within the loop regions, with the number of paired helical regions remaining constant (Liu and Huang 2010; Zhongying et al. 2020). Variations in rRNA between different species have been observed in both the loop and helical regions (Liu and Huang 2010). Owing to the compact nature of tRNA sequences, the secondary structures predicted on the basis of these sequences tend to be relatively accurate. Conversely, owing to the length of rRNA sequences, significant variability is observed in specific segments, which complicates the prediction of secondary structures and results in comparatively low levels of detected conservatism (Gruber et al. 2011).

### 4.3 Natural Selection Pressure in Paired and Unpaired Regions

The Tajima's D test results revealed that all the mtRNAs, whether in the paired or unpaired region, deviated from the neutral hypothesis in the evolutionary process (Tajima's *D* < 0). These findings suggest that these sequences have been subject to specific pressures of natural selection throughout their evolutionary history. Consequently, it can be inferred that these sequences are not completely random in their evolution but have undergone a nonrandom process influenced by natural selection (Tajima 1989). A more pronounced deviation from the neutral selection is exhibited by paired regions in a greater number of datasets (14 vs. 10), with a higher frequency of significant deviations from the neutral hypothesis being observed in paired regions than in their unpaired region (4 vs. 1, *p* < 0.05) (Table 1). This finding indicates that paired regions have been under stronger natural selection pressure than unpaired regions have. This finding supports the hypothesis that nonrandom evolutionary selection has played a greater role in maintaining the structural stability of paired regions (Cheng et al. 2012; Muhire et al. 2014).

Previous studies have demonstrated that compensatory evolution is driven by natural selection, which favors the stability of the thermodynamic structure. Compensatory substitution usually occurs in the helical region, which is crucial for RNA function (Cheng et al. 2012; Muhire et al. 2014; Tajima 1989). This phenomenon is particularly pronounced in tRNAs and is also observed in orthopterans (Table 1). In rRNAs, while both paired and unpaired regions are subject to purifying selection and show nonrandom patterns of evolution, there is limited evidence for natural selection pressure acting on the paired regions. This may be due to the use of a conservative general structure to delineate these regions, which may lead to inaccuracies in the representation of the secondary structures of individual sequences. Additionally, Tajima's D test has been shown to provide more reliable estimates for shorter sequences (Tajima 1989); however, it should be noted that rRNA sequences are typically longer.

### 4.4 Coevolution in mtRNAs

It has been observed that nucleotides within the RNA strand engage in paired interactions such that changes at one site can be compensated for only by corresponding changes in its interacting partners. This pairing has been remarkably conserved throughout evolutionary history, resulting in a pronounced pattern of coevolution (Cheng et al. 2012; Galtier and Dutheil 2007). These coevolutionary pressures arise from functional or structural selective pressures that act to maintain specific subsets of residues at these positions (Dutheil and Galtier 2007; Muhire et al. 2014; Talavera et al. 2015). A significant number of coevolutionary sites were also identified in Orthoptera mtRNAs, with the majority located within the mating region (Table 2, Figures S2–S4), providing robust support for previous findings. The complex interplay between sequence coevolution and secondary structure is a direct consequence of concerted efforts to preserve optimal structural and functional integrity (Cheng et al. 2012; Dib et al. 2014). Consequently, elucidating the intricacies of intramolecular coevolution is essential for interpreting the diverse structural and functional constraints imposed on RNA molecules. Such insight, in turn, facilitates predictions of molecular interactions and structures. Furthermore, an accurate understanding of the evolutionary patterns of RNA molecules will provide a theoretical basis for the use of RNA data to construct more accurate evolutionary relationships (Meyer et al. 2019).

### 4.5 Differences Between Replacement Models

A comparative analysis of the model parameters revealed consistent exchange rate patterns (4 × 4 matrix) between the four bases in the ring region across the three models (mtRNA16, mtRNA7, and mtRNA6). Moreover, there were no statistically significant differences in the parameters of the 4 × 4 matrix among these models (*p* > 0.1) (Figure 6). A similar trend of consistency was observed in the 6 × 6 replacement matrix of the six typical WC base pairs, and no significant differences in the parameters of this standard pairing matrix were found across all three models (*p* > 0.1) (Table S1). Additionally, no significant differences in the compositional frequencies of bases and base pairs were detected among the three state models (*p* > 0.1). The composition proportions of the four bases and the typical WC base pairs in the coding sequence followed a consistent order, suggesting that the three models are similar in terms of parameter changes, despite variations in matrix sizes. For the mtRNA16 model with a 20 × 20 matrix, there were significant differences in model parameters between the original Blosum62 model and the Orthopteria‐specific empirical alternative model mtOrt (*p* < 0.05). This finding suggests that the parameters of the new model differ from those in existing models and may offer some specificity.

To evaluate the robustness of the novel three‐state model derived from the training dataset, we constructed three distinct datasets containing 292, 93, and 199 species, respectively. Using the same methodology, model parameters for each of the three states were calculated from these datasets. A comparative analysis was then performed on the four model parameters for each state. The impact of varying data volumes (increasing/decreasing) and different taxonomic groups on model parameters was assessed to determine the robustness of the model trained on the original dataset. The results demonstrate that all three novel state models exhibit robustness (Table S4). Among them, the mtRNA16 model proved to be the most robust; its estimated parameters were minimally affected by changes in data volume or taxonomic composition. This indicates that the mtRNA16 model parameters, obtained from the training dataset, effectively fit Orthoptera data of varying sizes and taxonomic groups, accurately capturing the characteristics of the state‐16 dataset. The mtRNA7 model came second, while the mtRNA6 model was more significantly affected by data variations.

To make the substitution matrix in the model more representative, we used the training dataset containing a large amount of species data to calculate the model parameters. The data in the test dataset was not used for the construction of the model. The fit of the new model to the test dataset was evaluated through the fitting analysis of the new model and the data not involved in the modeling, because in actual analysis, the data used to build the tree is usually not entirely the same as the data used for model construction. The evaluation results of the mtRNA16, mtRNA7, and mtRNA6 models based on different state test datasets indicate that the mtRNA16 model provided a better fit to the 16‐state coding dataset than the original Blosum62 model and mtOrt did (Figure 7). While both mtRNA7 and mtRNA6 exhibited better performance in comparison with that of the existing model, mtOrt, when fitted to the 7‐ and 6‐state datasets, respectively, they did not reach the level of effectiveness demonstrated by mtRNA16. This finding indicates that the alternative 16‐state model has the capacity to effectively fit not only 16‐state datasets but also 7‐ and 6‐state datasets.

### 4.6 Comparison of Different Phylogenetic Trees

The parameters of six phylogenetic trees (mtRNA16_tree, mtRNA7_tree, mtRNA6_tree, GTR_tree, mtOrt_tree, and Best_tree) constructed based on different data and different models were compared. The results revealed that the branch support values (bootstrap values) of different trees exhibit a highly significant positive correlation (Table S5‐1, lower triangle, with most *r* > 0.95, *p* < 0.01), indicating that despite potential differences in topological structure, the patterns of high or low branch support are highly consistent across different tree‐building methods. The bootstrap values between mtRNA16_tree and Best_tree demonstrate a highly significant positive correlation (*r* = 0.970, *p* < 0.01), which is the highest bootstrap correlation with the optimal tree among the three new model trees (Table S5‐1). In contrast to the high consistency exhibited by bootstrap values, the correlations between site log‐likelihood values across different trees are generally low and highly variable (upper triangle of Table S5‐1). This is due to the use of different datasets and models in the construction of these trees, which reflects differences in the interpretation of patterns of sequence variation by these models. A comparison of the mtRNA6_tree with the Best_tree revealed that the former exhibited a negative correlation with the optimal tree in terms of site log‐likelihood values. This finding provides an indirect indication that the mtRNA6 model fits the data to a lesser extent than the other two new models. The GTR_tree exhibited the lowest correlation with the optimal tree in terms of site log‐likelihood values, thereby suggesting that the DNA model exhibits a poor fit for RNA sequences. The findings of the branch length analysis are, in general, consistent with those of the log‐likelihood values. The majority of branch length estimates demonstrate significant positive correlations (*p* < 0.01 or *p* < 0.05), yet the absolute values of correlation coefficients are typically modest (0.002–0.284). This finding suggests that branch length estimates are highly sensitive to the dataset and model employed (Table S5‐2). The differences in topological structure were assessed using standardized RF distances and MS distances (the normalized average distance obtained from the Yule model) (Bogdanowicz et al. 2012) (Table S5‐3). All RF and MS distances between tree pairs were found to be greater than zero, thereby confirming the hypothesis that the phylogenetic hypotheses generated by different methods exhibit substantial topological divergence (see Table S5‐3 for details). In comparison with the optimal tree, both RF and MS distances indicate that the topological distance between mtRNA16_tree and Best_tree is the smallest relative to other trees (RF = 0.263, MS = 0.181). By contrast, the distances between mtRNA6_tree/mtRNA7_tree and Best_tree are larger (RF = 0.361/0.345, MS = 0.290/0.322), which corresponds with the analysis results of branch support. This further emphasizes the reliability of the mtRNA16 tree construction results and the instability of the mtRNA6 and mtRNA7 tree construction results, as well as those based on DNA models and RNA sequences (Gesell and Schuster 2014; Hudelot et al. 2003; Telford et al. 2005).

Based on the visualization results, we conducted a detailed comparative analysis of the topological structures of different trees. The results showed that the topological structures of mtRNA7_tree and mtOrt_tree revealed their inability to accurately separate the two suborders of Orthoptera (Caelifera and Ensifera) into distinct major branches (Figures S7 and S8). This finding contrasts with previously established evolutionary relationships derived from molecular and morphological data (Chang, Nie, et al. 2020; Chang, Qiu, et al. 2020; Song et al. 2015; Zhang, Huang, et al. 2013). In other trees, Orthoptera was divided into two monophyletic groups, Caelifera and Ensifera, which are sister groups to each other (Figure 8, Figures S5 and S6). Consequently, the trees constructed from the RNA sequence data using these two models (mtRNA7 and mtOrt) lack reliability. In mtOrt_tree, not all six outgroups clustered at the root of the whole tree (Figure S8), whereas in mtRNA7_tree, *Lipotactes tripyrga* from Caelifera appeared as a single branch at the root of Orthoptera (Figure S7). Given these apparent errors in the branching position in both trees, we believe that their results are inaccurate; therefore, we will refrain from further discussion of the interbranch relationships within these two trees.

The primary distinctions among the Ensifera superfamilies center on the categorization of Rhaphidophoroidea and Stenopelmatoidea, which exhibit rearrangements in position. Notably, Rhaphidophoroidea is monophyletic in Best_tree (Figure S5), a finding that is consistent with the conclusions of previous studies that utilized mitogenome data (Song et al. 2015; Zhou et al. 2017). However, this pattern is not reflected in other phylogenetic trees. In this study, both the tree constructed using DNA sequences (GTR_tree) (Figure 8) and those derived from previous studies indicate that Stenopelmatoidea is monophyletic. Conversely, trees based on RNA and protein sequences fail to demonstrate monophyly (Figure 8, Figures S5 and S6). This finding indicates that not only do different models influence phylogenetic results, but the type of data utilized also exerts a substantial effect on the outcomes of tree construction (Chang, Nie, et al. 2020; Chang, Qiu, et al. 2020; Song et al. 2015; Zhou et al. 2017).

The distinguishing characteristics of the superfamily of Caelifera in different trees are primarily related to Pyrgomorphoidea, Pneumoroidea, and Tetrigoidea. The phylogenetic relationships among these superfamilies within Caelifera are fully consistent in both mtRNA16_tree and Best_tree (Figure S5), with a similar topological structure to that reported in previous studies (Chang, Nie, et al. 2020; Chang, Qiu, et al. 2020; Song et al. 2015; Zhang, Huang, et al. 2013). A positional shift of Pyrgomorphoidea is observed in the general time reversible (GTR) tree, where it clusters with Pamphagidae (Figure 8), a finding that contradicts previous phylogenetic trees constructed using the GTR model and DNA sequences (Chang et al. 2024; Chang, Qiu, et al. 2020; Song et al. 2015; Zhang, Huang, et al. 2013; Zhang, Zeng, et al. 2013). This finding suggests that reliance on mtRNA genes alone to elucidate the phylogeny of Orthoptera may result in somewhat inaccurate outcomes. Positional changes in Pneumoridae and Tetrigoidea were observed in mtRNA6_tree (Figure S6), but the parameters and structure of this tree indicate that mtRNA6_tree does not accurately represent the topology of Orthoptera. Moreover, the placements of these two suborders in mtRNA6_tree deviate from those reported in previous studies, as do those in the more robust mtRNA16_tree and Best_tree (Figure S5), suggesting that the positions of Pneumoroidea and Tetrigoidea within mtRNA6_tree lack reference values.

Across different phylogenetic trees, family positions exhibited greater variability (blue lines in Figure 8 and Figures S5–S8). The positional differences between families within Ensifera largely mirror those observed at the superfamily level, albeit with minor discrepancies between mtRNA16_tree, Best_tree, and GTR_tree (Figure 8 and Figure S5). The placement of Rhaphidophoridae and Gryllacrididae in these three trees is of particular interest, as it is not consistent across all three trees. Both mtRNA16_tree and Best_tree indicate that the initial divergence within the nongrylloid branch of Ensifera is represented by Gryllacrididae, a finding that is consistent with results derived from mitochondrial protein sequences and mtOrt (Chang, Nie, et al. 2020) but inconsistent with trees constructed using mitogenome sequences and GTR (Song et al. 2015; Zhou et al. 2017). In the grylloid branch, the relationships between families were consistent among mtRNA16_tree, Best_tree, and GTR_tree (Figure 8 and Figure S5). This finding is consistent with previous findings, which indicate that the topological structure of these families within the grylloid is relatively robust in all three phylogenetic trees (Chang, Nie, et al. 2020; Chang, Qiu, et al. 2020).

The differences in the topological structures of families within Caelifera across different phylogenetic trees were mainly observed for Episactidae, Tristiridae, Pyrgomorphidae, Pamphagidae, Ommexechidae, and Acrididae. Among these, Episactidae, Tristiridae, and Ommexechidae each include a single species, and their placement varied considerably between trees (Chang, Nie, et al. 2020; Chang, Qiu, et al. 2020; Song et al. 2015). Further data on related species are essential for a more comprehensive exploration of this issue. The monophyly of Pyrgomorphidae was supported in all trees, but the differences in their position in different trees were consistent with those of the superfamily Pyrgomorphoidea. Furthermore, a closer relationship with the superfamily Acridoidea is supported by previous studies (Chang et al. 2024; Chang, Qiu, et al. 2020; Song et al. 2015; Zhang, Zeng, et al. 2013). The family Pamphagidae (Acridoidea) is recognized as monophyletic and has a close phylogenetic relationship with the families Pyrgacrididae, Tristiridae, Lentulidae, Lithidiidae, and Ommexechidae. However, the actual relationships between these taxa remain unclear. mtRNA6_tree in particular presents a complex scenario (Figure S6), with significant discrepancies from other trees and extant research (Chang, Nie, et al. 2020; Chang, Qiu, et al. 2020; Song et al. 2015; Zhang, Huang, et al. 2013; Zhang, Zeng, et al. 2013). Moreover, the scarcity of mitochondrial genomic data available for the families Pyrgacrididae, Tristiridae, Lentulidae, Lithidiidae, Ommexechidae, and Romaleidae necessitates the acquisition of additional data to elucidate their phylogenetic relationships. The family Acrididae, recognized as the largest family of Acridoidea, has traditionally been considered monophyletic in previous studies. However, the incorporation of Dericorythidae into the phylogenetic framework of Acridoidea challenges this monophyly (Chang, Nie, et al. 2020). The relationships between subfamilies within Acrididae demonstrate significant variability across different phylogenetic trees. Relying solely on mitochondrial mtRNA data, mtRNA gene sequences, or RNA modeling is insufficient to establish a stable evolutionary relationship among Acrididae. A more diverse set of data and methods is essential for further exploration.

The construction of a phylogenetic tree from DNA datasets utilizing the GTR model has shown limited capacity in accurately resolving individual branch positions, a deficiency that is particularly pronounced in Acrididae. However, this approach exhibited an overall performance that is suboptimal in comparison to that of mtRNA16 but superior to that of mtRNA7 and mtRNA6 (Figure 8 and Figures S5–S8). Additionally, the application of mtOrt to construct trees on the basis of posttranscriptional mtRNA sequences also resulted in inaccurate outcomes (Figure S8). This inaccuracy can be attributed primarily to the fact that mtOrt is an empirical substitution model derived from mitochondrial protein sequences, which significantly differ from mtRNA data, rendering it unsuitable for the latter application. In summary, from the perspective of phylogenetic evolutionary relationships within Orthoptera, our analysis of new RNA models with three different states indicates that mtRNA16 achieves optimal performance, whereas mtRNA7 and mtRNA6 yield suboptimal results. This finding implies that empirical evolutionary models constructed using data that either uniformly account for mispairing or completely overlook mispairing information may lose critical phylogenetic evolutionary signals, resulting in inaccurate topological structures. Consequently, empirical RNA evolution models that comprehensively incorporate all secondary structure information can infer more accurate phylogenetic evolutionary relationships (Gesell and Schuster 2014; Hudelot et al. 2003; Meyer et al. 2019; Nasrallah et al. 2011; Smith et al. 2004; Subbotin et al. 2007; Telford et al. 2005).

Since one of the three newly sequenced mitochondrial genomes in this study lacked a *trnY* ( *T. bivittata* ), two additional datasets were constructed on the basis of the dataset containing 24 post‐encoded three‐state RNA sequences (including 24 RNA data, namely mtRNA6) from 298 species. The purpose of this was to test the impact of tRNA deletion on the results of the three new phylogenetic trees. One of the datasets involved the removal of the *trnY* data from all species (including 23 RNA data, namely mtRNA6‐23), while the other involved the random removal of the *trnY* data from one species (containing 24 RNA data but lacking the *trnY* data from one species, namely mtRNA6‐24‐1), resulting in six new data sets. The three new models were then employed for the construction of the phylogenetic trees, employing the same method as described earlier (Discussion 2.6). The analysis yielded a total of six phylogenetic trees, which were designated mtRNA16‐23_tree, mtRNA16‐24‐1_tree, mtRNA7‐23_tree, mtRNA7‐24‐1_tree, mtRNA6‐23_tree, and mtRNA6‐24‐1_tree, respectively. A comparison was then made between the six newly constructed trees and the three trees built using the dataset containing 24 RNA sequences and the three‐state model (mtRNA16_tree, mtRNA7_tree, and mtRNA6_tree). The results indicated that the site log‐likelihood values among the three trees constructed using the mtRNA16 model (mtRNA16_tree, mtRNA16‐23_tree and mtRNA16‐24‐1_tree), the three trees constructed using the mtRNA7 model (mtRNA7_tree, mtRNA7‐23_tree and mtRNA7‐24‐1_tree), and the three trees constructed using the mtRNA6 model (mtRNA6_tree, mtRNA6‐23_tree and mtRNA6‐24‐1_tree) were only correlated between the mtRNA16‐23_tree and mtRNA16‐24‐1_tree, with a correlation coefficient of 0.985, while all other trees had a value of 1 (Table S5‐4). Additionally, a highly significant correlation was identified between the log‐likelihood values of all trees (*p* < 0.01), with no significant differences observed (*p* > 0.05). The bootstrap values demonstrated high and significant correlations between disparate trees (*r* > 0.99, *p* < 0.01), thereby indicating that both site log‐likelihood values and bootstrap values exhibited a high degree of consistency among the three trees in the same state. The differences in branch length are not statistically significant (*p* > 0.05) in the three‐state trees. In the 16‐state trees, the highly significant positive correlations are observed (*r* > 0.92, *p* < 0.01). In the 7‐state and 6‐state trees, trees lacking tRNA data demonstrate a positive correlation with trees constructed using 24 RNA data, while others exhibit a negative correlation. However, none of these correlations are statistically significant. This finding indicates that the absence of tRNA data exerts a lesser influence on branch length in 16‐state trees, yet has a more pronounced effect on 7‐state and 6‐state trees. Topological distance analysis shows that the topological differences between 16‐state trees are smaller than those between 7‐state and 6‐state trees, regardless of whether MS or RF distance is used (Table S5‐5). Furthermore, the smallest topological distances are exhibited by trees constructed from datasets lacking *trnY* data for one species in the 16‐state trees, and by those constructed from 24 RNA datasets (MS =0.004, RF = 0.007). This phenomenon is not observed in 7‐state or 6‐state trees, which indicates that changes in tRNA data have the least impact on 16‐state trees. The 16‐state model better fits the data, whereas the 7‐state and 6‐state models are more sensitive to changes in the data during tree construction. A comparison of the visualization results reveals that there is only one branch difference between mtRNA16_tree and mtRNA16‐24‐1_tree and only three between mtRNA16_tree and mtRNA16‐23_tree, with most differences occurring at lower taxonomic levels. In contrast, there are more branch differences in the 7‐ and 6‐state trees, which is consistent with the statistical analysis results (Figure S9). These results further validate our previous finding that the 16‐state model, which incorporates more secondary structure information, better fits the data and provides more phylogenetic signals, thereby enhancing the reliability of phylogenetic analysis.

## 5 Conclusion

This study demonstrated that mtRNA secondary structures are universally conserved in Orthoptera, with stability maintained by coevolution driven by compensatory mutations under natural selection. By reencoding the four nucleotide‐based sequences into twenty symbol‐encoded datasets, we developed empirical substitution models (mtRNA16, mtRNA7, and mtRNA6) that reflect different approaches to handle base mispairing. Comparative analyses revealed that the mtRNA16 model, which incorporates all possible pairing scenarios, exhibits exceptional robustness and significantly outperforms both the mtRNA7 and mtRNA6 models, as well as traditional models such as the GTR and mtOrt models, in resolving phylogenetic relationships. This study, as an initial exploration of RNA empirical evolutionary models, emphasizes the importance of incorporating secondary structure information into evolutionary models. In the future, different types of data can be combined, and different evolutionary models can be applied to different data partitions to explore the phylogenetic relationships of Orthoptera. This will provide a more accurate framework for understanding the evolutionary dynamics of Orthoptera and potentially other taxa.

## Author Contributions

**Huihui Chang:** conceptualization (equal), data curation (equal), formal analysis (lead), funding acquisition (lead), investigation (equal), methodology (equal), visualization (lead), writing – original draft (lead), writing – review and editing (lead). **Yuan Huang:** conceptualization (equal), data curation (equal), investigation (equal), methodology (equal), writing – original draft (supporting), writing – review and editing (supporting). **Lina Zhao:** formal analysis (supporting), investigation (supporting), methodology (supporting). **Qianqian Liu:** formal analysis (supporting), visualization (supporting), writing – original draft (supporting). **Zhaohui Xie:** investigation (supporting), writing – original draft (supporting). **Nian Liu:** conceptualization (equal), data curation (equal), formal analysis (supporting), funding acquisition (lead), writing – original draft (supporting), writing – review and editing (supporting).

## Ethics Statement

The authors have nothing to report.

## Conflicts of Interest

The authors declare no conflicts of interest.

## Supporting information


**Data S1:** ece372068‐sup‐0001‐DataS1.docx.

**Data S2:** ece372068‐sup‐0002‐DataS2.xlsx.

## Data Availability

All data generated or analyzed during this study are included in this published article, its Supporting Information files, and publicly available repositories. Publicly available sequencing data, which were analyzed in this study, are listed in Table S1. The relevant data and code for this study are available on GitHub (https://github.com/huihuicahng/mtRNAs/tree/main).

## References

[ece372068-bib-0001] Akiyama, M. , and K. Sato . 2023. “RNA Secondary Structure Prediction Based on Energy Models.” Methods in Molecular Biology 2586: 89–105. 36705900 10.1007/978-1-0716-2768-6_6

[ece372068-bib-0002] Allen, J. E. , and S. Whelan . 2014. “Assessing the State of Substitution Models Describing Noncoding RNA Evolution.” Genome Biology and Evolution 6: 65–75. 24391153 10.1093/gbe/evt206 PMC3914692

[ece372068-bib-0004] Bernt, M. , A. Donath , F. Jühling , et al. 2013. “MITOS: Improved De Novo Metazoan Mitochondrial Genome Annotation.” Molecular Phylogenetics and Evolution 69: 313–319. 22982435 10.1016/j.ympev.2012.08.023

[ece372068-bib-0005] Bogdanowicz, D. , K. Giaro , and B. Wróbel . 2012. “TreeCmp: Comparison of Trees in Polynomial Time.” Evolutionary Bioinformatics 8: 475.

[ece372068-bib-0006] Byun, Y. , and K. Han . 2009. “PseudoViewer3: Generating Planar Drawings of Large‐Scale RNA Structures With Pseudoknots.” Bioinformatics 25: 1435–1437. 19369500 10.1093/bioinformatics/btp252

[ece372068-bib-0007] Caetano‐Anolles, G. 2002. “Tracing the Evolution of RNA Structure in Ribosomes.” Nucleic Acids Research 30: 2575–2587. 12034847 10.1093/nar/30.11.2575 PMC117177

[ece372068-bib-0008] Cameron, S. L. 2014. “Insect Mitochondrial Genomics: Implications for Evolution and Phylogeny.” Annual Review of Entomology 59: 95–117. 10.1146/annurev-ento-011613-162007 24160435

[ece372068-bib-0009] Cannone, J. J. , S. Subramanian , M. N. Schnare , et al. 2002. “The Comparative RNA Web (CRW) Site: An Online Database of Comparative Sequence and Structure Information for Ribosomal, Intron, and Other RNAs.” BMC Bioinformatics 3: 2. 11869452 10.1186/1471-2105-3-2 PMC65690

[ece372068-bib-0010] Carapelli, A. , F. N. Soto‐Adames , C. Simon , F. Frati , F. Nardi , and R. Dallai . 2004. “Secondary Structure, High Variability and Conserved Motifs for Domain III of 12S rRNA in the Arthropleona (Hexapoda; Collembola).” Insect Molecular Biology 13: 659–670. 15606814 10.1111/j.0962-1075.2004.00528.x

[ece372068-bib-0011] Chang, H. , X. Guo , S. Guo , N. Yang , and Y. Huang . 2021. “Trade‐Off Between Flight Capability and Reproduction in Acridoidea (Insecta: Orthoptera).” Ecology and Evolution 11: 16849–16861. 34938477 10.1002/ece3.8317 PMC8668762

[ece372068-bib-0012] Chang, H. , X. Liu , and Z. Xie . 2024. “The Complete Mitochondrial Genome of *Phymateus Saxosus* (Coquerel, 1861) (Orthoptera: Pyrgomorphidae) and Phylogenetic Analysis.” Mitochondrial DNA Part B Resources 9: 457–460. 38591051 10.1080/23802359.2024.2316064 PMC11000610

[ece372068-bib-0013] Chang, H. , Y. Nie , N. Zhang , et al. 2020. “MtOrt: An Empirical Mitochondrial Amino Acid Substitution Model for Evolutionary Studies of Orthoptera Insects.” BMC Evolutionary Biology 20: 57. 32429841 10.1186/s12862-020-01623-6 PMC7236349

[ece372068-bib-0014] Chang, H. , Z. Qiu , H. Yuan , et al. 2020. “Evolutionary Rates of and Selective Constraints on the Mitochondrial Genomes of Orthoptera Insects With Different Wing Types.” Molecular Phylogenetics and Evolution 145: 106734. 31972240 10.1016/j.ympev.2020.106734

[ece372068-bib-0015] Cheng, N. , Y. H. Mao , Y. Y. Shi , and S. H. Tao . 2012. “Coevolution in RNA Molecules Driven by Selective Constraints: Evidence From 5S rRNA.” PLoS One 7: e44376. 22973441 10.1371/journal.pone.0044376 PMC3433437

[ece372068-bib-0016] Cigliano, M. M. , H. Braun , D. C. Eades , and D. Otte . 2025. “Orthoptera Species File. Version 5.0/5.0.” http://Orthoptera.SpeciesFile.org.

[ece372068-bib-0017] Clary, D. O. , and D. R. Wolstenholme . 1987. “ *Drosophila* Mitochondrial DNA: Conserved Sequences in the A + T‐Rich Region and Supporting Evidence for a Secondary Structure Model of the Small Ribosomal RNA.” Journal of Molecular Evolution 25: 116–125. 3116271 10.1007/BF02101753

[ece372068-bib-0018] Crooks, G. E. , G. Hon , J. M. Chandonia , and S. E. Brenner . 2004. “WebLogo: A Sequence Logo Generator.” Genome Research 14: 1188–1190. 15173120 10.1101/gr.849004 PMC419797

[ece372068-bib-0019] Dang, C. C. , V. S. Le , O. Gascuel , B. Hazes , and Q. S. Le . 2014. “FastMG: A Simple, Fast, and Accurate Maximum Likelihood Procedure to Estimate Amino Acid Replacement Rate Matrices From Large Data Sets.” BMC Bioinformatics 15: 1–10. 25344302 10.1186/1471-2105-15-341 PMC4287512

[ece372068-bib-0020] Dib, L. , D. Silvestro , and N. Salamin . 2014. “Evolutionary Footprint of Coevolving Positions in Genes.” Bioinformatics 30: 1241–1249. 24413673 10.1093/bioinformatics/btu012

[ece372068-bib-0021] Dierckxsens, N. , P. Mardulyn , and G. Smits . 2017. “NOVOPlasty: De Novo Assembly of Organelle Genomes From Whole Genome Data.” Nucleic Acids Research 45: e18. 28204566 10.1093/nar/gkw955 PMC5389512

[ece372068-bib-0022] Do, C. B. , D. A. Woods , and S. Batzoglou . 2006. “CONTRAfold: RNA Secondary Structure Prediction Without Physics‐Based Models.” Bioinformatics 22: e90–e98. 16873527 10.1093/bioinformatics/btl246

[ece372068-bib-0023] Dutheil, J. , and N. Galtier . 2007. “Detecting Groups of Coevolving Positions in a Molecule: A Clustering Approach.” BMC Evolutionary Biology 7: 242. 18053141 10.1186/1471-2148-7-242 PMC2248193

[ece372068-bib-0024] Dutheil, J. Y. 2012. “Detecting Coevolving Positions in a Molecule: Why and How to Account for Phylogeny.” Briefings in Bioinformatics 13: 228–243. 21949241 10.1093/bib/bbr048

[ece372068-bib-0025] Dutheil, J. Y. , F. Jossinet , and E. Westhof . 2010. “Base Pairing Constraints Drive Structural Epistasis in Ribosomal RNA Sequences.” Molecular Biology and Evolution 27: 1868–1876. 20211929 10.1093/molbev/msq069

[ece372068-bib-0026] Eddy, S. R. 2011. “Accelerated Profile HMM Searches.” PLoS Computational Biology 7: e1002195. 22039361 10.1371/journal.pcbi.1002195 PMC3197634

[ece372068-bib-0027] Galtier, N. , and J. Dutheil . 2007. “Coevolution Within and Between Genes.” Genome Dynamics 3: 1–12. 18753781 10.1159/000107599

[ece372068-bib-0028] Gesell, T. , and P. Schuster . 2014. “Phylogeny and Evolution of RNA Structure.” In RNA Sequence, Structure, and Function: Computational and Bioinformatic Methods, edited by J. Gorodkin and W. L. Ruzzo , 319–378. Humana Press. 10.1007/978-1-62703-709-9_16 24639167

[ece372068-bib-0029] Gillespie, J. J. , J. S. Johnston , J. J. Cannone , and R. R. Gutell . 2006. “Characteristics of the Nuclear (18S, 5.8S, 28S and 5S) and Mitochondrial (12S and 16S) rRNA Genes of *Apis mellifera* (Insecta: Hymenoptera): Structure, Organization, and Retrotransposable Elements.” Insect Molecular Biology 15: 657–686. 17069639 10.1111/j.1365-2583.2006.00689.x PMC2048585

[ece372068-bib-0030] Golden, M. , B. Murrell , D. Martin , O. G. Pybus , and J. Hein . 2020. “Evolutionary Analyses of Base‐Pairing Interactions in DNA and RNA Secondary Structures.” Molecular Biology and Evolution 37: 576–592. 31665393 10.1093/molbev/msz243 PMC6993869

[ece372068-bib-0031] Grant, J. R. , E. Enns , E. Marinier , et al. 2023. “Proksee: In‐Depth Characterization and Visualization of Bacterial Genomes.” Nucleic Acids Research 51: W484–W492. 37140037 10.1093/nar/gkad326 PMC10320063

[ece372068-bib-0032] Grimaldi, D. , and M. S. Engel . 2005. Evolution of the Insects. Cambridge University Press.

[ece372068-bib-0033] Gruber, A. R. , S. Findei , S. Washietl , I. L. Hofacker , and P. F. Stadler . 2011. “RNAz 2.0: Improved Noncoding RNA Detection.” Pacific Symposium on Biocomputing Pacific Symposium on Biocomputing 15: 69–79. 19908359

[ece372068-bib-0034] Hickson, R. E. , C. Simon , A. Cooper , G. S. Spicer , J. Sullivan , and D. Penny . 1996. “Conserved Sequence Motifs, Alignment, and Secondary Structure for the Third Domain of Animal 12S rRNA.” Molecular Biology and Evolution 13: 150–169. 8583888 10.1093/oxfordjournals.molbev.a025552

[ece372068-bib-0035] Higgs, G. P. 2001. “RNA Secondary Structure: Physical and Computational Aspects.” Quarterly Reviews of Biophysics 33: 199–253. 10.1017/s0033583500003620 11191843

[ece372068-bib-0036] Higgs, P. G. 1998. “Compensatory Neutral Mutations and the Evolution of RNA.” Genetica 102: 91–101. 9720274

[ece372068-bib-0037] Hofacker, I. L. 2003. “Vienna RNA Secondary Structure Server.” Nucleic Acids Research 31: 3429–3431. 12824340 10.1093/nar/gkg599 PMC169005

[ece372068-bib-0038] Hudelot, C. , V. Gowri‐Shankar , H. Jow , M. Rattray , and P. G. Higgs . 2003. “RNA‐Based Phylogenetic Methods: Application to Mammalian Mitochondrial RNA Sequences.” Molecular Phylogenetics and Evolution 28: 241–252. 12878461 10.1016/s1055-7903(03)00061-7

[ece372068-bib-0039] Kalyaanamoorthy, S. , B. Q. Minh , T. K. F. Wong , A. von Haeseler , and L. S. Jermiin . 2017. “ModelFinder: Fast Model Selection for Accurate Phylogenetic Estimates.” Nature Methods 14: 587–589. 28481363 10.1038/nmeth.4285 PMC5453245

[ece372068-bib-0040] Katoh, K. , G. Asimenos , and H. Toh . 2009. “Multiple Alignment of DNA Sequences With MAFFT.” Methods in Molecular Biology 537: 39–64. 19378139 10.1007/978-1-59745-251-9_3

[ece372068-bib-0041] Kearse, M. , R. Moir , A. Wilson , et al. 2012. “Geneious Basic: An Integrated and Extendable Desktop Software Platform for the Organization and Analysis of Sequence Data.” Bioinformatics 28: 1647–1649. 22543367 10.1093/bioinformatics/bts199 PMC3371832

[ece372068-bib-0042] Kerpedjiev, P. , S. Hammer , and I. L. Hofacker . 2015. “Forna (Force‐Directed RNA): Simple and Effective Online RNA Secondary Structure Diagrams.” Bioinformatics 31: 3377–3379. 26099263 10.1093/bioinformatics/btv372 PMC4595900

[ece372068-bib-0043] Kosakovsky Pond, S. L. , F. V. Mannino , M. B. Gravenor , S. V. Muse , and S. D. Frost . 2007. “Evolutionary Model Selection With a Genetic Algorithm: A Case Study Using Stem RNA.” Molecular Biology and Evolution 24: 159–170. 17038448 10.1093/molbev/msl144

[ece372068-bib-0044] Lan, S. , Y. Ma , X. Pu , R. Ji , and L. Yuan . 2023. “Complete Mitochondrial Genome and Phylogenetic Analysis of *Oedaleus decorus decorus* .” Genomics and Applied Biology 42: 832–844.

[ece372068-bib-0045] Lian, D. , J. Wei , X. Ding , Y. Liu , and Q. Zhao . 2022. “Comparison and Application of tRNA and rRNA Genes in the Mitochondrial Genome of Pentatomidae (Hemiptera: Pentatomoidea).” Joumal of Fujian Agriculture and Forestry University 51: 782–791.

[ece372068-bib-0046] Liu, N. , and Y. Huang . 2010. “Complete Mitochondrial Genome Sequence of *Acrida cinerea* (Acrididae: Orthoptera) and Comparative Analysis of Mitochondrial Genomes in Orthoptera.” Comparative and Functional Genomics 2010: 319486. 21197069 10.1155/2010/319486 PMC3004375

[ece372068-bib-0047] Liu, Y. , F. Song , P. Jiang , J. J. Wilson , W. Cai , and H. Li . 2018. “Compositional Heterogeneity in True Bug Mitochondrial Phylogenomics.” Molecular Phylogenetics and Evolution 118: 135–144. 28986237 10.1016/j.ympev.2017.09.025

[ece372068-bib-0048] Meyer, X. , L. Dib , D. Silvestro , and N. Salamin . 2019. “Simultaneous Bayesian Inference of Phylogeny and Molecular Coevolution.” Proceedings of the National Academy of Sciences of the United States of America 116: 5027–5036. 30808804 10.1073/pnas.1813836116 PMC6421416

[ece372068-bib-0049] Misof, B. , and G. Fleck . 2003. “Comparative Analysis of Mt LSU rRNA Secondary Structures of Odonates: Structural Variability and Phylogenetic Signal.” Insect Molecular Biology 12: 535–548. 14986915 10.1046/j.1365-2583.2003.00432.x

[ece372068-bib-0050] Muhire, B. M. , M. Golden , B. Murrell , et al. 2014. “Evidence of Pervasive Biologically Functional Secondary Structures Within the Genomes of Eukaryotic Single‐Stranded DNA Viruses.” Journal of Virology 88: 1972–1989. 24284329 10.1128/JVI.03031-13 PMC3911531

[ece372068-bib-0051] Muse, S. V. 1995. “Evolutionary Analyses of DNA Sequences Subject to Constraints on Secondary Structure.” Genetics 139: 1429–1439. 7768450 10.1093/genetics/139.3.1429 PMC1206468

[ece372068-bib-0052] Nasrallah, C. A. , D. H. Mathews , and J. P. Huelsenbeck . 2011. “Quantifying the Impact of Dependent Evolution Among Sites in Phylogenetic Inference.” Systematic Biology 60: 60–73. 21081481 10.1093/sysbio/syq074 PMC2997629

[ece372068-bib-0053] Nguyen, L. T. , H. A. Schmidt , A. von Haeseler , and B. Q. Minh . 2015. “IQ‐TREE: A Fast and Effective Stochastic Algorithm for Estimating Maximum‐Likelihood Phylogenies.” Molecular Biology and Evolution 32: 268–274. 25371430 10.1093/molbev/msu300 PMC4271533

[ece372068-bib-0054] Pollock, D. D. , W. R. Taylor , and N. Goldman . 1999. “Coevolving Protein Residues: Maximum Likelihood Identification and Relationship to Structure.” Journal of Molecular Biology 287, no. 1: 187–198. 10074416 10.1006/jmbi.1998.2601

[ece372068-bib-0055] Ren, L. , X. Zhang , Y. Li , et al. 2020. “Comparative Analysis of Mitochondrial Genomes Among the Subfamily Sarcophaginae (Diptera: Sarcophagidae) and Phylogenetic Implications.” International Journal of Biological Macromolecules 161: 214–222. 32526299 10.1016/j.ijbiomac.2020.06.043

[ece372068-bib-0056] Renée, E. , and M. Tillier . 1994. “Maximum Likelihood With Multiparameter Models of Substitution.” Journal of Molecular Evolution 39: 409–417.

[ece372068-bib-0057] Rousset, F. , M. Pélandakis , and M. Solignac . 1991. “Evolution of Compensatory Substitutions Through G.U Intermediate State in Drosophila rRNA.” Proceedings of the National Academy of Sciences of the United States of America 88: 10032–10036. 1946420 10.1073/pnas.88.22.10032 PMC52861

[ece372068-bib-0058] Rozas, J. , A. Ferrer‐Mata , J. C. Sanchez‐DelBarrio , et al. 2017. “DnaSP 6: DNA Sequence Polymorphism Analysis of Large Data Sets.” Molecular Biology and Evolution 34: 3299–3302. 29029172 10.1093/molbev/msx248

[ece372068-bib-0059] Savill, N. J. , D. C. Hoyle , and P. G. Higgs . 2001. “RNA Sequence Evolution With Secondary Structure Constraints: Comparison of Substitution Rate Models Using Maximum‐Likelihood Methods.” Genetics 157: 399–411. 11139520 10.1093/genetics/157.1.399 PMC1461489

[ece372068-bib-0060] Schöniger, M. , and A. Von Haeseler . 1994. “A Stochastic Model for the Evolution of Autocorrelated DNA Sequences.” Molecular Phylogenetics and Evolution 3: 240–247. 7529616 10.1006/mpev.1994.1026

[ece372068-bib-0061] Shah, R. A. , M. Riyaz , S. Ignacimuthu , and K. Sivasankaran . 2022. “Characterization of Four Mitochondrial Genomes From Superfamilies Noctuoidea and Hyblaeoidea With Their Phylogenetic Implications.” Scientific Reports 12: 18926. 36344589 10.1038/s41598-022-21502-y PMC9640664

[ece372068-bib-0062] Smith, A. D. , T. W. Lui , and E. R. Tillier . 2004. “Empirical Models for Substitution in Ribosomal RNA.” Molecular Biology and Evolution 21: 419–427. 14660689 10.1093/molbev/msh029

[ece372068-bib-0063] Song, H. 2018. “Biodiversity of Orthoptera.” In Insect Biodiversity, 245–279. Wiley‐Blackwell.

[ece372068-bib-0064] Song, H. , C. Amdgnato , M. M. Cigliano , et al. 2015. “300 Million Years of Diversification: Elucidating the Patterns of Orthopteran Evolution Based on Comprehensive Taxon and Gene Sampling.” Cladistics 31: 621–651. 34753270 10.1111/cla.12116

[ece372068-bib-0065] Song, H. , O. Bethoux , S. Shin , et al. 2020. “Phylogenomic Analysis Sheds Light on the Evolutionary Pathways Towards Acoustic Communication in Orthoptera.” Nature Communications 11: 4939. 10.1038/s41467-020-18739-4 PMC7532154 33009390

[ece372068-bib-0066] Subbotin, S. A. , D. Sturhan , N. Vovlas , et al. 2007. “Application of the Secondary Structure Model of rRNA for Phylogeny: D2‐D3 Expansion Segments of the LSU Gene of Plant‐Parasitic Nematodes From the Family Hoplolaimidae Filipjev, 1934.” Molecular Phylogenetics and Evolution 43: 881–890. 17101282 10.1016/j.ympev.2006.09.019

[ece372068-bib-0067] Tahi, F. , T. T. V. Du , and A. Boucheham . 2017. “In Silico Prediction of RNA Secondary Structure.” In Methods in Molecular Biology, vol. 1543, 145–168. Humana Press. 28349425 10.1007/978-1-4939-6716-2_7

[ece372068-bib-0068] Tajima, F. 1989. “Statistical Method for Testing the Neutral Mutation Hypothesis by DNA Polymorphism.” Genetics 123: 585–595. 2513255 10.1093/genetics/123.3.585 PMC1203831

[ece372068-bib-0069] Talavera, D. , S. C. Lovell , and S. Whelan . 2015. “Covariation Is a Poor Measure of Molecular Coevolution.” Molecular Biology and Evolution 32: 2456–2468. 25944916 10.1093/molbev/msv109 PMC4540965

[ece372068-bib-0070] Tang, F. , Y. Zhang , and Y. Zhao . 2017. “Morphological and Molecular Identification of the New Species, *Trichodina pseudoheterodentata* sp. n. (Ciliophora, Mobilida, Trichodinidae) From the Channel Catfish, *Ictalurus punctatus* , in Chongqing China.” Journal of Eukaryotic Microbiology 64: 45–55. 27253201 10.1111/jeu.12335

[ece372068-bib-0071] Telford, M. J. , M. J. Wise , and V. Gowri‐Shankar . 2005. “Consideration of RNA Secondary Structure Significantly Improves Likelihood‐Based Estimates of Phylogeny: Examples From the Bilateria.” Molecular Biology and Evolution 22: 1129–1136. 15689526 10.1093/molbev/msi099

[ece372068-bib-0072] Tillier, E. R. M. , and R. A. Collins . 1998. “High Apparent Rate of Simultaneous Compensatory Base‐Pair Substitutions in Ribosomal RNA.” Genetics 148: 1993–2002. 9560412 10.1093/genetics/148.4.1993 PMC1460107

[ece372068-bib-0073] Verma, C. , G. Mishra , and Omkar . 2020. “Widespread Inspection and Comparative Analysis of *ITS* Secondary Structure Conservation and Covariation of Coccinellidae.” International Journal of Tropical Insect Science 40: 587–597.

[ece372068-bib-0074] Wang, P. , F. Gao , J. Huang , M. Strüder‐Kypke , and Z. Yi . 2015. “A Case Study to Estimate the Applicability of Secondary Structures of SSU‐rRNA Gene in Taxonomy and Phylogenetic Analyses of Ciliates.” Zoologica Scripta 44: 574–585.

[ece372068-bib-0075] Wolstenholme, D. R. 1992. “Animal Mitochondrial DNA: Structure and Evolution.” In International Review of Cytology, edited by R. W. David and W. J. Kwang , 173–216. Academic Press. 10.1016/s0074-7696(08)62066-5 1452431

[ece372068-bib-0076] Yue, B.‐S. , H. Li , Y. Yan , and J. Li . 2023. “Eighteen Mitochondrial Genomes of Syrphidae (Insecta: Diptera: Brachycera) With a Phylogenetic Analysis of Muscomorpha.” PLoS One 18: e0278032. 36602958 10.1371/journal.pone.0278032 PMC9815649

[ece372068-bib-0077] Zhang, C. , B. Mao , H. Wang , et al. 2023. “The Complete Mitogenomes of Three Grasshopper Species With Special Notes on the Phylogenetic Positions of Some Related Genera.” Insects 14: 85. 36662013 10.3390/insects14010085 PMC9865218

[ece372068-bib-0078] Zhang, D. , F. L. Gao , I. Jakovlic , et al. 2020. “PhyloSuite: An Integrated and Scalable Desktop Platform for Streamlined Molecular Sequence Data Management and Evolutionary Phylogenetics Studies.” Molecular Ecology Resources 20: 348–355. 31599058 10.1111/1755-0998.13096

[ece372068-bib-0080] Zhang, H. L. , H. H. Zeng , Y. Huang , and Z. M. Zheng . 2013. “The Complete Mitochondrial Genomes of Three Grasshoppers, *Asiotmethis zacharjini*, *Filchnerella helanshanensis* and *Pseudotmethis rubimarginis* (Orthoptera: Pamphagidae).” Gene 517: 89–98. 23291499 10.1016/j.gene.2012.12.080

[ece372068-bib-0081] Zhang, H. L. , L. Zhao , Z. M. Zheng , and Y. Huang . 2013. “Complete Mitochondrial Genome of *Gomphocerus sibiricus* (Orthoptera: Acrididae) and Comparative Analysis in Four Gomphocerinae Mitogenomes.” Zoological Science 30: 192–204. 23480379 10.2108/zsj.30.192

[ece372068-bib-0079] Zhang, H.‐L. , Y. Huang , L.‐L. Lin , X.‐Y. Wang , and Z.‐M. Zheng . 2013. “The Phylogeny of the Orthoptera (Insecta) as Deduced From Mitogenomic Gene Sequences.” Zoological Studies 52: 1–13.

[ece372068-bib-0082] Zhongying, Q. , C. Huihui , Y. Hao , et al. 2020. “Comparative Mitochondrial Genomes of Four Species of *Sinopodisma* and Phylogenetic Implications (Orthoptera, Melanoplinae).” Zookeys 969: 23–42. 33013166 10.3897/zookeys.969.49278 PMC7515930

[ece372068-bib-0083] Zhou, Z. , L. Zhao , N. Liu , et al. 2017. “Towards a Higher‐Level Ensifera Phylogeny Inferred From Mitogenome Sequences.” Molecular Phylogenetics and Evolution 108: 22–33. 28188878 10.1016/j.ympev.2017.01.014

