# Supplementary material for: Developing and Applying RNA Empirical Models With Secondary Structure Insights for Orthoptera Phylogenetics
Source: Ecol Evol. 2025 Aug 31;15(9):e72068. doi: 10.1002/ece3.72068 (PMC12399576; doi:10.1002/ece3.72068)
Supplement: Supplementary file 1 — Data S1: ece372068‐sup‐0001‐DataS1.docx. [file ECE3-15-e72068-s001.docx]

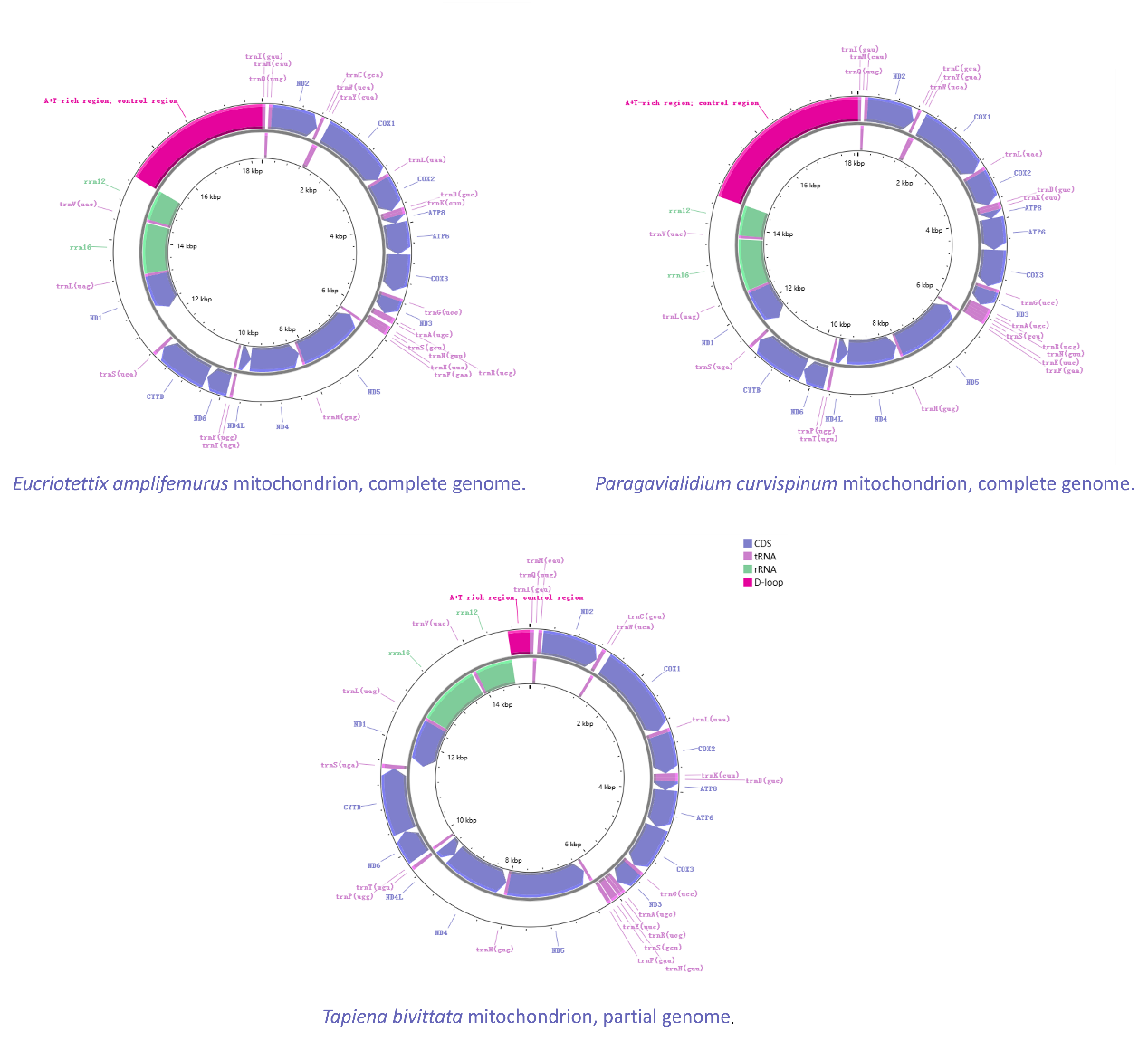
Fig. S1 Gene map of the mitochondrial genomes of *Eucriotettix amplifemurus*, *Paragavialidium curvispinum* and *Tapiena bivittata*


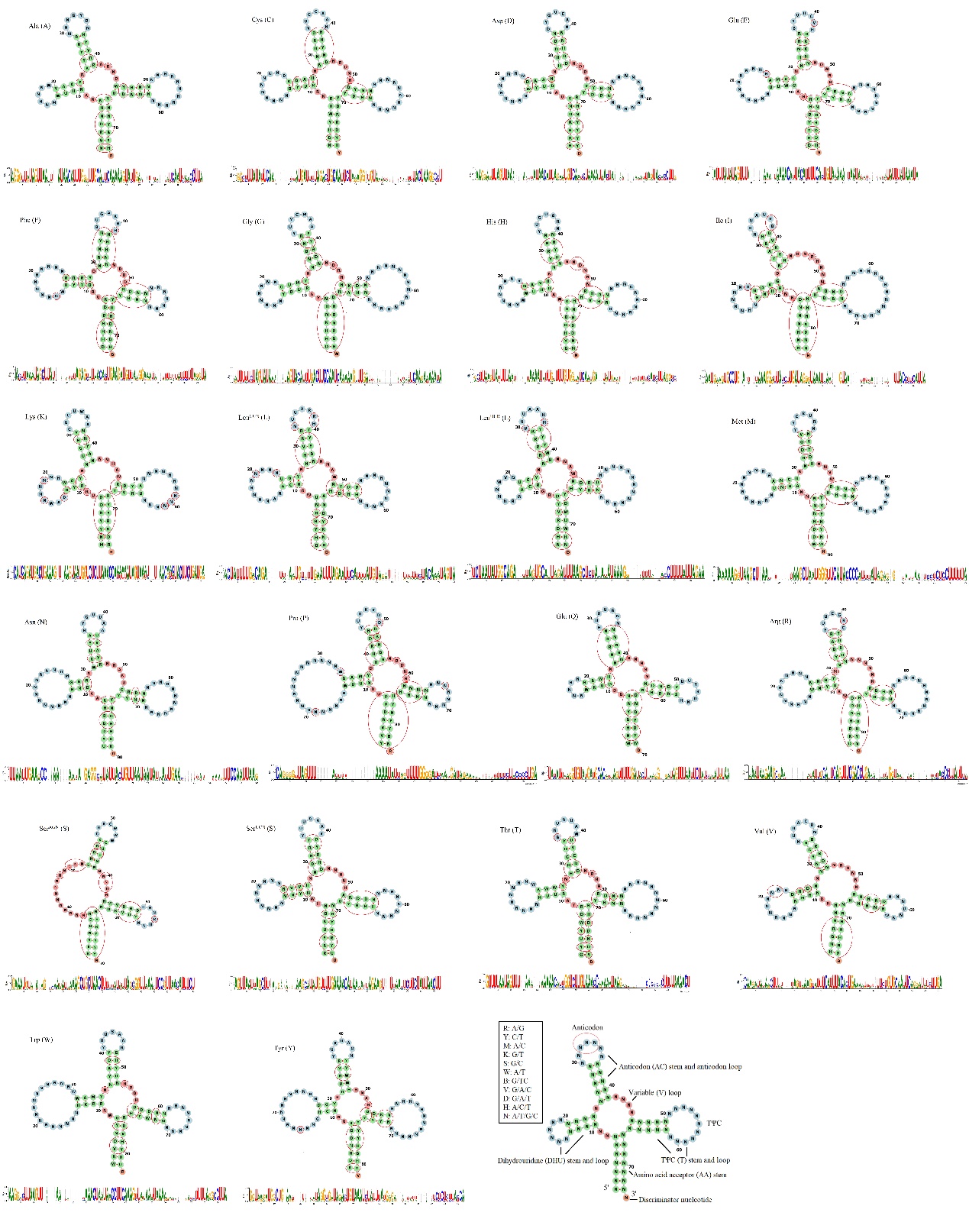


Fig. S2 Secondary structure diagram of the consensus sequence of 22 tRNAs in the Orthoptera mitochondrial genome. The sites circled in red are coevolution sites


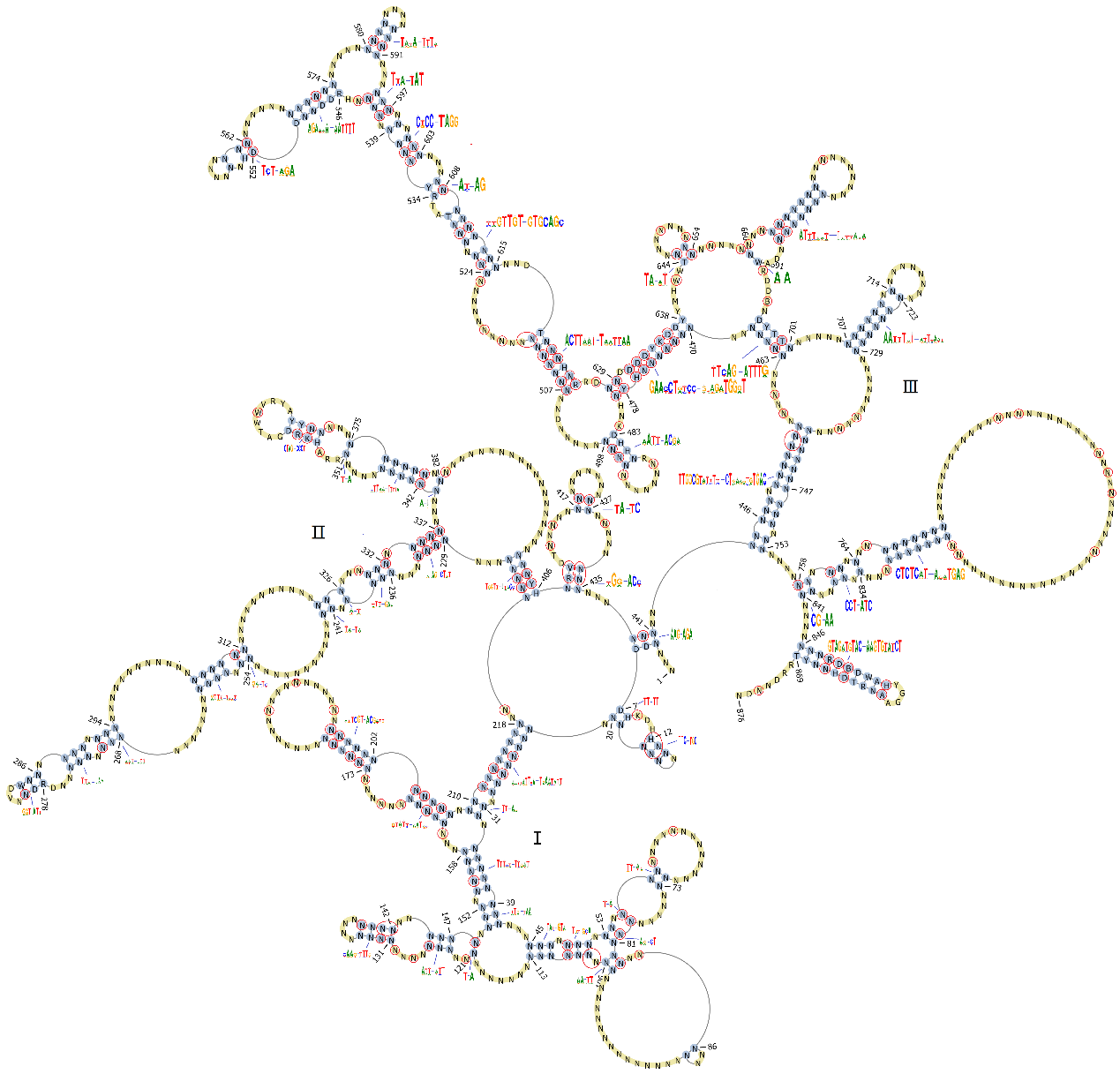


Fig. S3 Secondary structure diagram of the consensus sequence of *rrnS* in the Orthoptera mitochondrial genome. The sites circled in red are coevolution sites


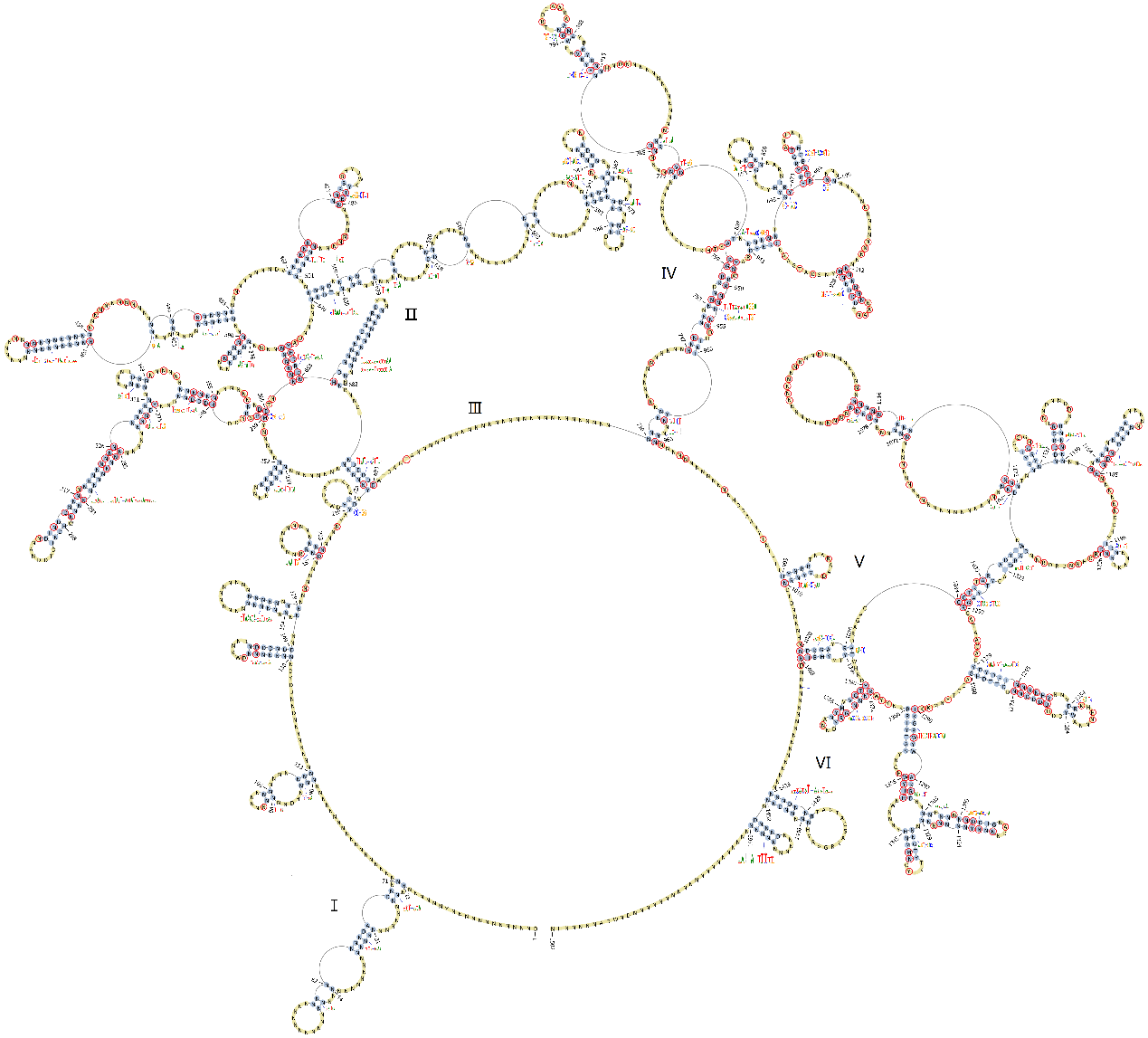


Fig. S4 Secondary structure diagram of the consensus sequence of *rrnL* in the Orthoptera mitochondrial genome. The sites circled in red are coevolution sites


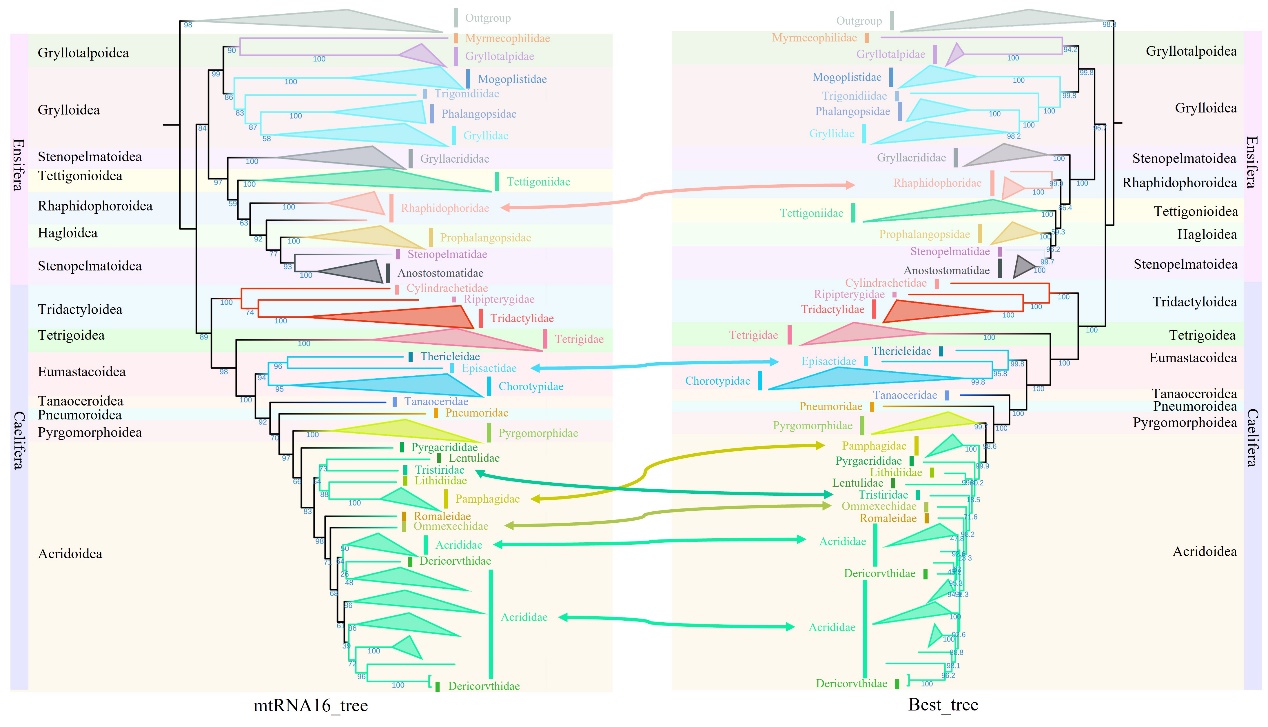


Fig. S5 The phylogenetic relationships among the higher taxa of Orthoptera in mtRNA16_tree and Best_tree. The leftmost/rightmost branches of the two trees, the background colour, and the colour of the branches represent the superfamilies. Different families are distinguished by different colours. The arrows between the two trees indicate the branches where the two trees diverge, and the arrow colours represent different families.


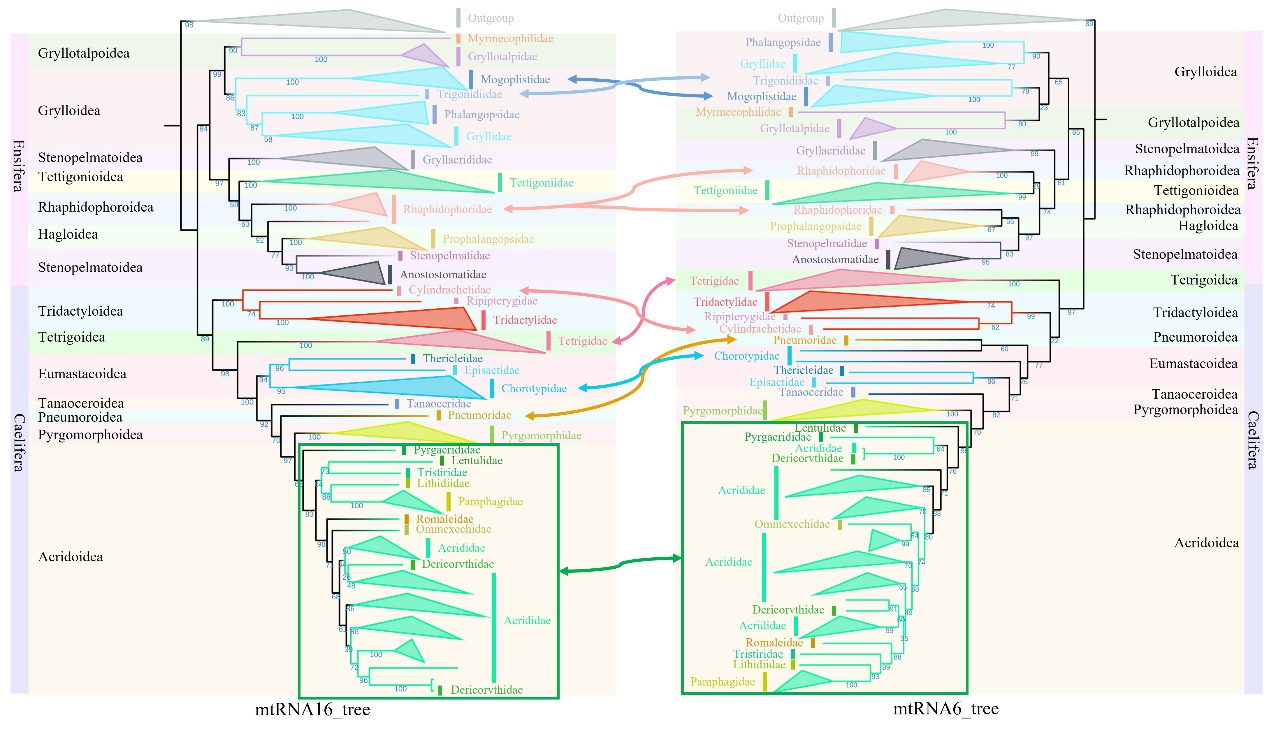


Fig. S6 The phylogenetic relationships among the higher taxa of Orthoptera in mtRNA16_tree and mtRNA6_tree. The leftmost/rightmost branches of the two trees, the background colour, and the colour of the branches represent the superfamilies. Different families are distinguished by different colours. The arrows between the two trees indicate the branches where the two trees diverge, and the arrow colours represent different families.


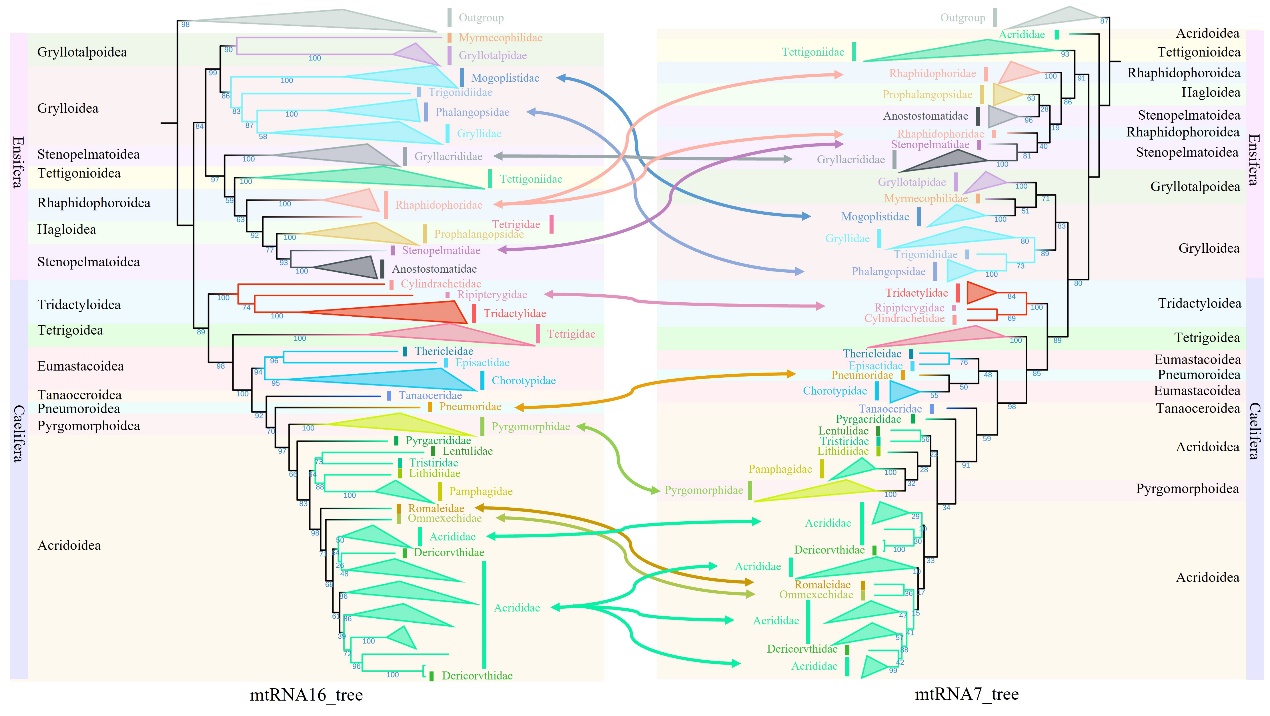


Fig. S7 The phylogenetic relationships among the higher taxa of Orthoptera in mtRNA16_tree and mtRNA7_tree. The leftmost/rightmost branches of the two trees, the background colour, and the colour of the branches represent the superfamilies. Different families are distinguished by different colours. The arrows between the two trees indicate the branches where the two trees diverge, and the arrow colours represent different families.


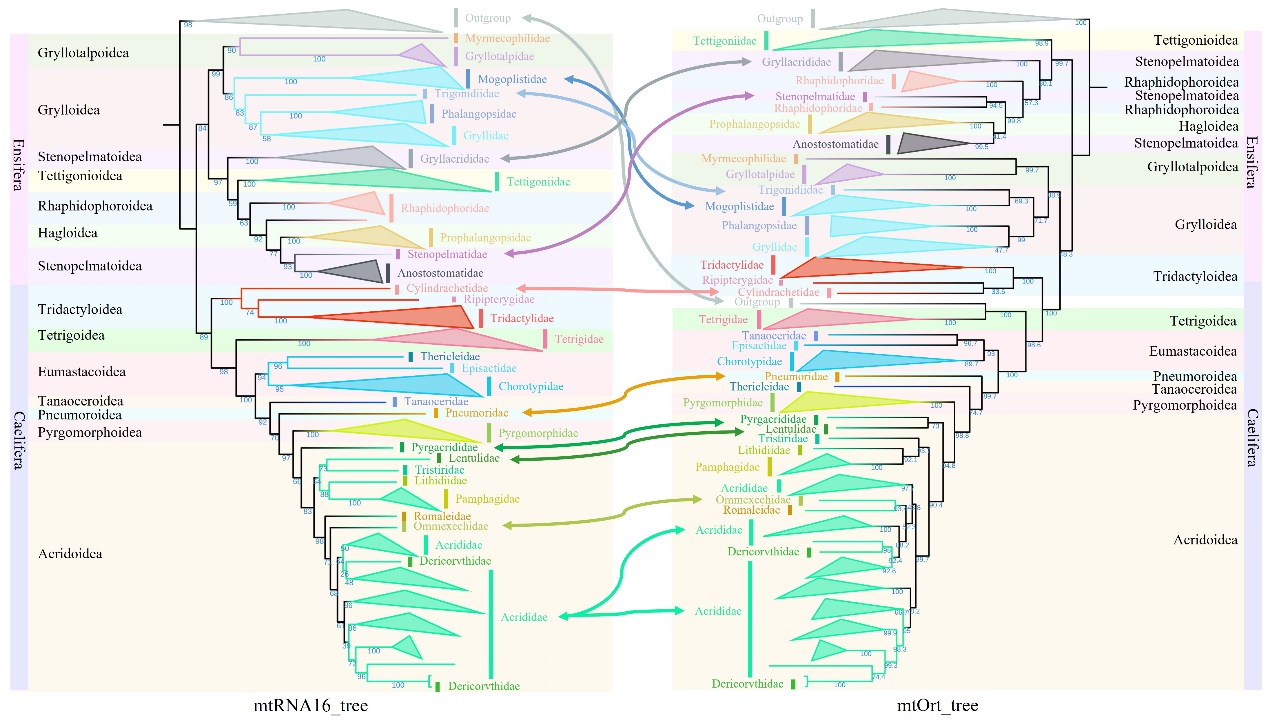


Fig. S8 The phylogenetic relationships among the higher taxa of Orthoptera in mtRNA16_tree and mtOrt_tree. The leftmost/rightmost branches of the two trees, the background colour, and the colour of the branches represent the superfamilies. Different families are distinguished by different colours. The arrows between the two trees indicate the branches where the two trees diverge, and the arrow colours represent different families.


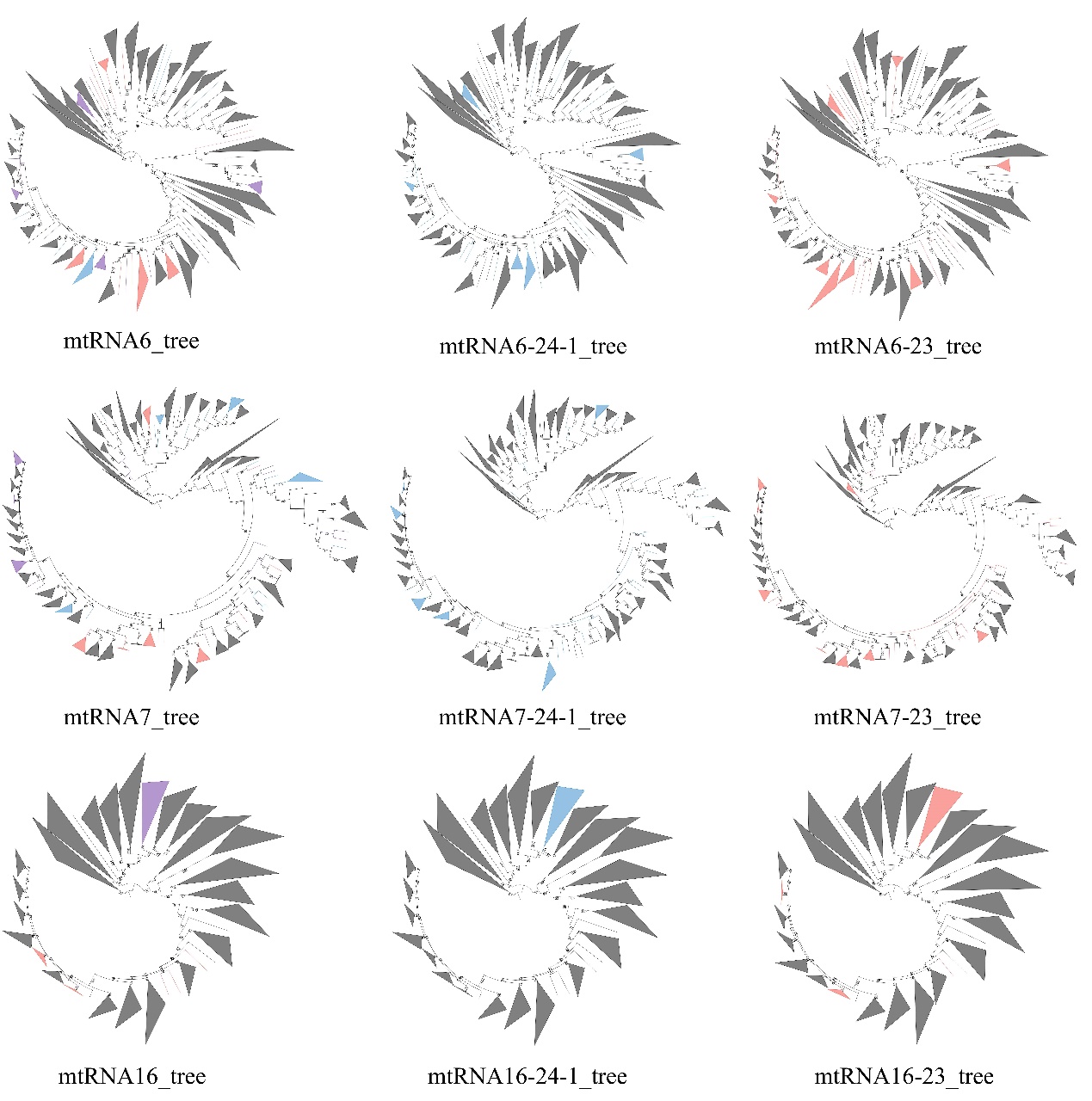


Fig. S9 Comparison of phylogenetic trees constructed using different datasets and three new models. The same model was used for each row of trees. The first column shows three trees (mtRNA16_tree, mtRNA7_tree, and mtRNA6_tree) constructed using the dataset containing 24 mitochondrial RNA recoding data from 298 species and three new models (mtRNA16, mtRNA7, and mtRNA6). The second column employs a dataset in which the *trnY* data for a single species has been randomly removed from the dataset presented in the first column. The third column employs a dataset in which the *trnY* data for all species has been randomly removed from the first column's dataset.
